# Evaluation of the Spatial Variations in the Biochemical Composition of Seaweed Species Along the Coast of Alexandria, With a Focus on Fatty Acids and Total Amino Acids of the Prevalent Edible Species

**DOI:** 10.1002/fsn3.70645

**Published:** 2025-07-16

**Authors:** Mona M. Ismail, José M. Miranda Lopez, Abeer A. M. El‐Sayed

**Affiliations:** ^1^ National Institute of Oceanography and Fisheries (NIOF) Cairo Egypt; ^2^ Laboratorio de Higiene, Inspección y Control de Alimentos (LHICA), Departamento de Química Analítica, Nutrición y Bromatología Universidade de Santiago de Compostela Lugo Spain

**Keywords:** algae, amino acids, niacin, special pigments, vitamin

## Abstract

The biochemical constituents of common seaweed (16 different species) from the coast of Alexandria, Egypt, were investigated, and the fatty acid and total amino acid contents of the three most dominant dietary species were assessed. *Ulva fasciata* has the highest moisture content (78.0% ± 9.9% fresh weight) in El Mex Bay, whereas in Gleem, *Jania rubens* has the greatest ash content (28.2% ± 2.9% dry weight), and *Pterocladia capillacea* has a high carbohydrate content (52.5% ± 5.2% DW). A high protein content was observed in *U. linza* (Abu‐Qir). In general, the lipid content of most seaweeds is low, and the caloric content of the selected species is also low at ≈5 kcal/100 g, making it a viable alternative biosource of healthy food to help combat obesity. *U. linza* and *Cladophora glomerata* are distinguished by their high photosynthetic pigments. *Padina boryana* has the highest fucoxanthin content. The highest level (5.26% ± 0.74%) of PUFA was found only in *P. capillacea,* indicating that it has the best nutritional indices. *P. boryana* and *P. capillacea* presented high essential amino acid (EAA)/NEAA ratios (0.65 ± 0.35 and 0.63 ± 0.69, respectively). Lysine is the limiting amino acid in *P. boryana,* with a low amino acid score (AAS) (62.20% ± 0.44%*). Padina boryana* and *P. capillacea* had EAAI values of 154% ± 0.95% and 122% ± 0.86%, respectively. Seaweed examined and their biochemical compositions, fatty acids, and total amino acids have the potential to be used in biofuel, medicine, cosmetics, and nutrition.

## Graphical abstract

Seaweeds from Alexandra are rich in vitamins E and B3, pigments, and total fatty acids. *Ulva linza* and *Cladophora glomerata* were distinguished by their high photosynthetic pigments. Amino acid score of all seaweeds investigated was > 100%.

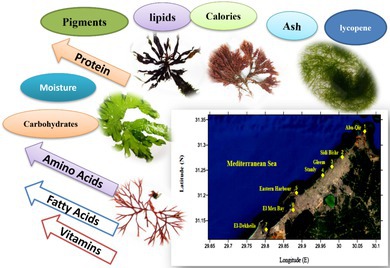

## 1 Introduction

Macroalgae, commonly known as seaweed, are crucial components of marine ecology and play an essential role in sustaining living organisms. They are large marine benthic algae that are multicellular, macrothallic, polyphyletic, and therefore distinct from most microscopic algae (microalgae). They are among the most commercially important marine renewable resources. Seaweeds are classified into three categories, namely, green algae (Chlorophyceae), red algae (Rhodophyceae), and brown algae (Phaeophyceae), on the basis of the pigments responsible for their color (Lopez‐Santamarina et al. 2020). There are an estimated 1800 different species of green seaweed, 6200 red seaweed, and 1800 brown seaweed in the marine environment (Zhong et al. 2020).

Although seaweed composition can vary depending on the species, habitat, season, maturity, and environmental conditions (Ismail, Elkomy, and El‐Sheekh 2023; Ito and Hori 1989), seaweeds are of nutritional interest because they are low‐calorie foods rich in minerals, proteins, polyphenols, polysaccharides, and dietary fiber, whereas their fat content is low (Ismail, El Zokm, and Miranda 2023). They are also a natural source of water‐soluble and fat‐soluble vitamins, such as thiamine, riboflavin, β‐carotene, and tocopherol (Ismail et al. 2016; Senapati et al. 2016). Additionally, other compounds from seaweed, such as gelatin, chitosan, phenolic compounds, or polysaccharides, are of interest for the pharmaceutical, medical, cosmetic, nutraceutical, food, and agricultural industries (Ismail, Elkomy, and El‐Sheekh 2023; Olasehinde et al. 2019; Shobier et al. 2023). Therefore, the short‐term goal of functional foods, nutraceuticals, and dietary supplements should be to ensure a high quality of life and enhance health status, whereas the long‐term goal should be to increase the life span (Shipeng et al. 2015). Thus, biochemical analysis of seaweed is crucial for assessing its nutritional value to marine herbivores and identifying potential sources of protein, carbohydrates, and lipids for human consumption or commercial use (Ismail et al. 2017; El Zokm et al. 2021).

A wide variety of seaweeds grow along the Egyptian Mediterranean coast, especially in Alexandria. Some studies have investigated the variations in the bioactive or chemical compositions of different seaweeds collected from certain locations along the Alexandria coast (El Zokm et al. 2021; Ismail et al. 2017). However, there have been no studies concerning the spatial variations in macroalgal biochemical composition.

Therefore, this study aimed to provide an overview of the spatial variation in the biochemical composition, including total carbohydrates, proteins, pigments, and vitamins, of common algal species along the Alexandria coast in Egypt. Statistical methods were applied to illustrate the significant variation between the different species. This included evaluating the fatty acids of the dominant species (*P. boryana*, *P. capillacea*, and *U. fasciata* ), with a focus on their nutritional value using different indices such as the Unsaturation index (UI), Atherogenic index (AI), Thrombogenic index (TI), and Hypocholesterolemic/Hypercholesterolemic ratio (H/H): unsaturation; atherogenic; thrombogenic; and Hypocholesterol emic/Hypercholesterolemic. Additionally, the total amino acid composition of the three selected species will be assessed through the amino acid score (AAS) and the essential amino acid index (EAAI) to highlight their nutritional values.

## 2 Materials and Methods

### 2.1 Collection of Macroalgal Species

**FIGURE 1:**
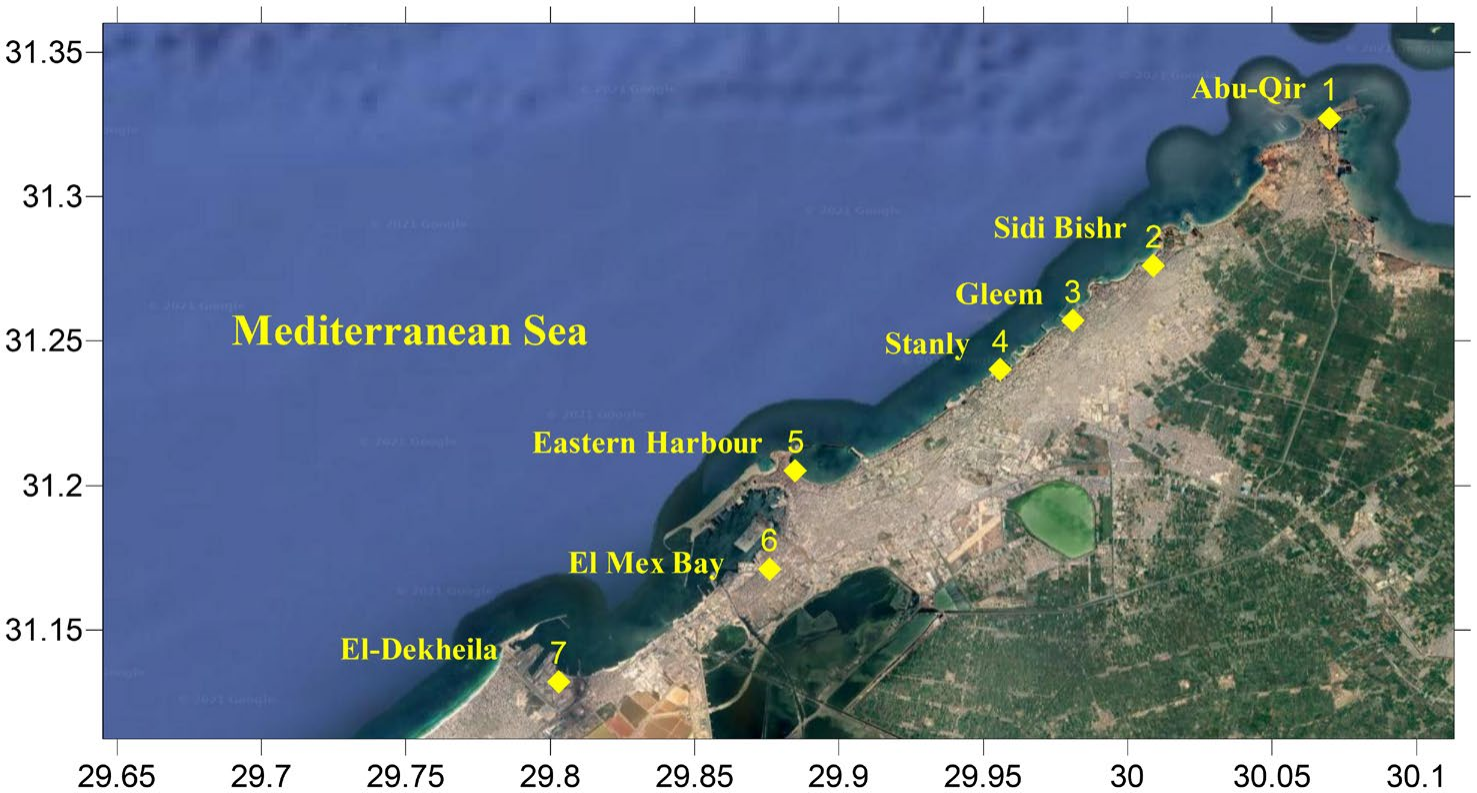
Sampling locations along Alexandria Coast.

The tested seaweed species were handpicked in spring 2023 at depths ranging from 0.5 to 1 m from seven sites (1–7) along the Alexandria coast by a square metal frame of 4 m^2^ (2 m × 2 m) (Table 1 and Figure 1). The selected samples were thoroughly cleaned with distilled water and a soft brush to remove residues and epiphytes. A portion of the fresh seaweed samples were processed as herbarium samples, while the remaining samples were preserved in 5% formalin for taxonomical identification by the National Institute of Oceanography and Fisheries, Alexandria (NIOF, Alexandria, Egypt), and confirmed by the Algae Base website (Guiry and Guiry 2022). The remaining pieces were air dried in the shade at ambient temperature, crushed into a fine powder, and stored at −20°C for further use.

**TABLE 1:** Sampling sites description along Alexandria coast.

Location	ID	Latitude (N)	Longitude (E)
Abu‐Qir	1	31°19′11″	30°03′28″
Sidi Bishr	2	31°16′07″	29°59′09″
Gleem	3	31°23′96″	29°96′03″
Stanly	4	31°14′02″	29°56′45″
Eastern Harbor (E.H)	5	31°12′19″	29°52′34″
El Mex Bay	6	31°09′04″	29°50′29″
El‐Dekheila (E.D)	7	31°07′22″	29°49′04″

### 2.2 Biochemical Analysis

The moisture content was estimated by drying in an oven between 100°C and 105°C according to the Association of Official Analytical Chemists (2022) technique. Marsham et al. (2007) approach was utilized to determine the ash content of the algae after 10 h at 550°C. The total lipid content was identified via the Association of Official Analytical Chemists (2022) assay. The total carbohydrate content was determined following the method outlined by Dubois et al. (1956). The total soluble protein content was measured via the method described by Lowry et al. (1951). These parameters are expressed as percentages of the seaweed dry weight. The caloric content was calculated via the following formula:

CaloreVauekcal/100gDW=4×protein%+9×Carbohydrate%+4×Lipid%

The vitamin (C, E and B_3_) contents of the tested algae were also determined. The Pantelidis et al. (2007) method was utilized to evaluate the vitamin C (ascorbic acid) content, with the results represented as mg ascorbic acid (AA) per 100 g fresh weight (FW). The vitamin E (α‐tocopherol) concentration was estimated via the Prieto et al. (1999) method and expressed as mg α‐tocopherol equivalents per gram of algal extract. Niacin (vitamin B3) was detected according to Nasreen et al. (2022) and expressed as mg/100 g dry weight (DW).

Photosynthetic pigments, including chlorophylls *a*, total chlorophylls, carotenoids, β‐carotene, and lycopene, were also determined. Chlorophyll *a* and total chlorophyll in the algal acetone extracts (90%) were determined via Connan's (2015) assay and estimated via the formula below and expressed as mg/g fresh weight:

$$\text{Chlorophylla}\;\left(\mathrm{mg}/\mathrm{gFW}\right)=\frac{\left[12.7\left(A663\right)-2.69\left(A645\right)\right]}{\text{Weight of the sample}}\times \text{extraction volume}$$

$$\text{Totalchlorophyll content}\;\left(\mathrm{mg}/\mathrm{gFW}\right)=\frac{\left[20.2\left(A645\right)+8.02\left(A663\right)\right]}{\text{Weight of the sample}}\times \text{extraction volume}$$

The carotenoid contents were determined according to the methods of Amorim‐Carrilho et al. (2014) and were calculated via the following formulation:

$$\text{Carotenoids}\;\left(\mathrm{mg}/\mathrm{gFW}\right)=\frac{4\times 480\times \text{extraction volume}}{\mathrm{Wt}.\text{of sample}}$$

Where A_480_, A_645_, and A_663_ = absorbance values at 480, 645, and 663 nm, respectively.

*β*‐Carotene and lycopene contents were measured in a mixed solvent of acetone–hexane according to Nagata and Yamashita (1992) via the following equations:

$$\beta -\text{Carotene}\;\left(\mathrm{mg}/100\right)=0.216\times A663-0.304\times A505+0.452\times A453$$

$$\text{Lycopen}\;\left(\mathrm{mg}/100g\right)=\left(-40.0458\times A663\right)+186\times \left(0.372\times A505\right)-\left(0.0806\times A453\right)$$

### 2.3 Accessory Pigments

Phycobiliproteins are water‐soluble pigment proteins that are unique to red algal species (Zhao et al. 2011). Phycobiliproteins (phycoerythrin [PE], allophycocyanine [APC], and phycocyanin [PC]) were extracted from the algae via phosphate buffer (0.1 M) at pH 6.8 (Pagdett and Krogman 1987) and were calculated as mg/g FW via the following formula:

$$\mathrm{PC}\;\left(\mathrm{mg}/\mathrm{gFW}\right)=\frac{\left(A615\right)\times \left(0.475\times A652\right)}{5.34}$$

$$\mathrm{APC}\;\left(\mathrm{mg}/\mathrm{gFW}\right)=\frac{\left(A652\right)\times \left(0.208\times A615\right)}{5.09}$$

$$\mathrm{PE}\;\left(\mathrm{mg}/\mathrm{gFW}\right)=\frac{\left(A562\right)-\left(2.41\times \mathrm{PC}\right)-\left(0.849\times \mathrm{APC}\right)}{9.62}$$

Where A_562_, A_615_, and A_652_ = absorbance values at 562, 615, and 652 nm, respectively.

Fucoxanthin is found in brown algae, and its concentration is determined by extracting fresh algal species in a mixture of dimethyl sulfoxide (DMSO) and water (4:1, v/v). The concentration of the extracted pigments was determined via the equation provided by Osório et al. (2020) and expressed as mg/g FW.

$$\text{Fucoxanthin}\;\left(\mathrm{mg}/\mathrm{gFW}\right)=\left[7.69-\left(A480-A750\right)-5.5\right]$$

where A_480_ and A_750_ are the absorbance values at 480 and 750 nm, respectively.

### 2.4 Fatty Acid Determination

The fatty acid contents of the collected seaweed samples were analyzed via gas chromatography (GC), specifically an HP (Hewlett Packard) 6890 GC with a flame ionization detector. The fatty acid content was measured by comparing the area to the internal standard and represented as a percentage of total fatty acid methyl esters (FAME).

### 2.5 Amino Acid Composition

The amino acid composition of the seaweed samples was determined via HPLC according to Cohen et al. (1989), as described by White et al. (1986) and Cohen et al. (1989). Before the sample was injected, the instrument was calibrated by two injections of lysine standards. The amino acid content was expressed as mg/g total amino acids (TAAs).

### 2.6 Nutritional Values

The equations for the nutritional indicators (Table S1) were based on the fatty acid content (Chen and Liu 2020).

### 2.7 Data Statistical Analysis

All the analytical measurements were performed in triplicate (*n* = 3), and the mean results were obtained. Statistica software version 12 (TIBCO Software Inc., Palo Alto, CA, USA) was used to analyze the data. The nonparametric Kruskal‐Wallis test was used to evaluate significant differences in biochemical composition among seaweed groups. Additionally, theKruskal‐Wallis test was performed to assess whether location had an effect on the biochemical composition of the seaweed. One‐way analysis of variance (ANOVA) was used to analyze the significant variation between the different biochemical parameters and pigment data in different locations at *p* < 0.05.

## 3 Results and Discussion

### 3.1 Biochemical Analysis

Tables S2–S29 shows that the Kruskal‐Wallis test revealed that green seaweeds had significantly the highest moisture (H (2, *N* = 51) = 34.83, *p* = 0.00), Chl a (H (2, *N* = 51) = 8.01, *p* = 0.02), and protein (H (2, *N* = 51) = 9.27, *p* = 0.01) contents. Additionally, red seaweeds had significantly the highest ash composition (H (2, *N* = 51) = 10.38, *p* = 0.01). However, other biochemical constituents did not show significant variations among groups of seaweeds (*p* > 0.05). Moreover, the sites did not have a significant effect on the biochemical composition of different groups of seaweeds (*p* > 0.05). The biochemical compositions (moisture, ash, total fat, carbohydrates, protein, and calories) of seaweed from different beaches are shown in Table 2. *U. fasciata* from El Mex Bay has the highest moisture content (78% ± 9.9% FW), whereas the red macroalga *J. longifurca* from Abu‐Qir has the lowest moisture content (28.5% ± 1.4% FW). According to previous works, moisture concentrations in seaweed can vary depending on the temperature, salinity, and cell membrane type (Ismail et al. 2017). The variation in ash content can be attributed to various factors, including algal type, seasonality, environmental factors, geographical location, physiological variations, and mineralization methods (Ismail et al. 2017; Ismail, El Zokm, and Miranda 2023). In the present work, *J. rubens* from the Gleem coast had the highest ash value (28.2% ± 2.9% DW), whereas the green alga *U. linza* from Abu‐Qir had the lowest content (3.25% ± 0.1% DW). Variations in ash content can be attributed to various factors, including algal type, seasonality, environmental factors, geographical location, physiological variations, and mineralization methods (Ismail et al. 2017; Ismail, El Zokm, and Miranda 2023). The brown species ( *P. boryana* ) collected from the Abu‐Qir region presented the lowest percentage of lipids (1.19% ± 0.03% DW), whereas *C. officinalis* from the Sidi Bishr district presented the highest value (7.9% ± 1.7% DW). The environment, harvesting time, habitat, and production region could contribute to the variance in lipid content (Ismail et al. 2017). The morphological makeup of each species, including the thickness and growth of the algae thallus, phycocolloid content, metabolic preferences, and photosynthetic activity, affects the amount of carbohydrates in the collected species. This percentage ranged from 34.6% ± 3.6% DW in *G. opuntioides* in the El Mex area to 52.5% ± 5.2% DW in *P. capillacea* in Gleem (El‐Sayed and Ismail 2022; Ismail et al. 2017).

**TABLE 2:** Biochemical compositions of the tested algal species from along Alexandria coast.

Allocation/Seaweed spp.	Group	Moisture % FW	Ash % DW	Lipid % DW	Carbohydrates % DW	Protein % DW	Calorie (kcal/100 g)
**Abu Qir Bay**							
*Dictyota dichotoma* (Hudson) Lamouroux	Brown	59.9 ± 2.2^b^	6.53 ± 0.4^d^	4.08 ± 0.4^b^	50.8 ± 2.5^a^	26.7 ± 1.1^c^	5.80 ± 0.5
*Dictyota linearis* (C. Agardh) Greville		52.4 ± 2.4^b^	6.17 ± 0.3^d^	3.98 ± 0.3^b^	48.5 ± 2.2^a^	30.5 ± 1.6^b^	5.74 ± 0.8
*Padina boryana* Thivy		58.3 ± 2.3^b^	5.25 ± 0.2^d^	1.19 ± 0.03 ^d^	42.1 ± 2.1^b^	25.8 ± 0.9^c^	4.87 ± 0.1^b^
*Padina pavonia* (Linnaeus) J.V. Lamouroux		60.3 ± 3.8^a^	7.35 ± 0.5^d^	2.43 ± 0.1^c^	50.8 ± 2.8^a^	25.9 ± 0.8^c^	5.71 ± 0.5^a^
*Padina tetrastromatica* Hauck		66.8 ± 3.5^a^	6.89 ± 0.4^d^	2.96 ± 0.2^c^	50.8 ± 2.9^a^	24.4 ± 0.6^c^	5.67 ± 0.9^a^
*Codium decorticatum* (Woodward) M.A. Howe	Green	70.3 ± 3.9^a^	3.25 ± 0.1^d^	2.35 ± 0.1^c^	52.2 ± 3.0^a^	25.8 ± 0.7^c^	5.82 ± 0.2^a^
*Ulva fasciata* Delile		68.6 ± 3.7^a^	5.92 ± 0.6^d^	3.50 ± 0.3^c^	42.7 ± 2.6^b^	38.1 ± 1.8^a^	5.51 ± 0.3^a^
*Ulva lactuca* Linnaeus		69.3 ± 2.3^a^	5.69 ± 0.4^d^	3.32 ± 0.4^c^	47.6 ± 2.8^a^	36.0 ± 1.8^a^	5.86 ± 0.7^a^
*Ulva linza* Linnaeus		72.3 ± 3.9^a^	3.25 ± 0.1^d^	2.46 ± 0.3^c^	50.1 ± 2.1^a^	43.0 ± 2.1^a^	6.33 ± 1.2^a^
*Amphiroa rigida* J.V. Lamouroux	Red	34.3 ± 1.2^c^	26.5 ± 1.2^a^	1.51 ± 0.04^d^	42.3 ± 2.4^b^	26.8 ± 0.9^c^	4.94 ± 0.8^b^
*Chondracanthus acicularis* (Roth) Fredericq		34.6 ± 1.9^c^	6.73 ± 0.4^d^	1.64 ± 0.04^d^	49.3 ± 2.8^a^	27.7 ± 0.9^c^	5.61 ± 0.9^a^
*Corallina officinalis* Linnaeus		38.3 ± 1.8^c^	26.8 ± 1.6^a^	2.16 ± 0.1^c^	40.6 ± 2.0^b^	31.6 ± 1.6^b^	5.0 ± 0.8 ^b^
*Jania rubens* (Linnaeus) Lamouroux		29.9 ± 1.6^d^	27.3 ± 1.7^a^	1.75 ± 0.06^d^	50.3 ± 2.9^a^	29.9 ± 1.4^b^	5.79 ± 1.1^a^
*Jania longifurca* Zanardini		28.5 ± 1.4^d^	27.2 ± 1.2^a^	3.70 ± 0.4^b^	50.1 ± 3.1^a^	30.9 ± 1.9^b^	5.89 ± 1.2^a^
*Polysiphona elongata* (Hudson) Sprengel		45.3 ± 2.5^c^	10.9 ± 0.7^c^	1.43 ± 0.03^d^	46.2 ± 2.6^b^	25.3 ± 0.8^c^	5.23 ± 1.1^b^
*Pterocladia capillacea* (Gmelin) Bornet		37.9 ± 2.3^c^	6.73 ± 0.5^d^	6.56 ± 0.6^a^	50.4 ± 2.9^a^	41.4 ± 2.8^a^	6.45 ± 1.0 ^b^
**Sidi Bishr station**							
*Ulva fasciata* Delile	Green	68.9 ± 4.5^a^	9.30 ± 0.8^c^	1.25 ± 0.4^d^	40.5 ± 4.6^b^	37.1 ± 2.9^a^	5.18 ± 0.2^b^
*Ulva linza* Linnaeus		66.0 ± 3.9^a^	20.9 ± 2.2^a^	2.20 ± 0.8^c^	39.0 ± 3.7^b^	36.1 ± 2.0^a^	5.04 ± 0.4^b^
*Pterocladia capillacea* (Gmelin) Bornet	Red	29.9 ± 1.9^d^	13.3 ± 1.3^b^	6.80 ± 0.9^a^	39.6 ± 3.06^b^	33.3 ± 1.7^b^	5.17 ± 0.2^b^
*Amphiroa rigida* J.V. Lamouroux		29.0 ± 1.9^d^	13.3 ± 1.0^b^	7.9 ± 1.7^a^	50.2 ± 5.6^a^	30.7 ± 1.0^b^	6.06 ± 0.5^a^
*Jania rubens* (Linnaeus) Lamouroux		30.3 ± 2.1^d^	15.3 ± 1.5^b^	5.00 ± 0.5^b^	51.7 ± 5.9^a^	38.2 ± 2.6^a^	6.38 ± 0.8^a^
*Corallina officinalis* Linnaeus		38.3 ± 2.8^c^	5.25 ± 0.4^d^	7.0 ± 1.4^a^	50.2 ± 5.8^a^	32.5 ± 1.9^b^	6.09 ± 0.7^a^
**Gleem station**							
*Padina boryana* Thivy	Brown	55.3 ± 4.2^b^	4.25 ± 0.2^d^	6.37 ± 1.1^a^	44.0 ± 2.9^b^	30.2 ± 3.7^b^	5.42 ± 0.5^b^
*Ulva fasciata* Delile	Green	65.4 ± 6.3^a^	7.25 ± 0.8^d^	1.77 ± 0.05	46.2 ± 3.4^b^	24.9 ± 2.1^c^	5.22 ± 0.4^b^
*Corallina officinalis* Linnaeus	Red	40.3 ± 3.9^c^	13.6 ± 2.8^b^	1.62 ± 0.03^d^	39.8 ± 2.1^b^	35.3 ± 3.0^a^	5.06 ± 0.3 ^b^
*Pterocladia capillacea* (Gmelin) Bornet		35.6 ± 2.7^c^	8.64 ± 1.5^d^	2.84 ± 0.8^c^	40.9 ± 3.0^b^	26.9 ± 2.8^c^	4.87 ± 0.2 ^b^
*Jania rubens* (Linnaeus) Lamouroux		42.3 ± 2.9^c^	9.25 ± 1.6^c^	7.14 ± 0.6^a^	50.9 ± 4.9^a^	27.0 ± 1.9^c^	5.95 ± 0.4 ^a^
*Amphiroa rigida* J.V. Lamouroux		35.7 ± 3.5^c^	11.3 ± 2.0^c^	2.10 ± 0.7^c^	42.5 ± 2.2^b^	27.0 ± 2.8^c^	4.99 ± 0.5 ^b^
*Corallina elongata* J. Ellis & Solander		36.3 ± 2.3^c^	9.85 ± 1.9^c^	1.51 ± 0.04^d^	46.2 ± 3.2^b^	32.5 ± 3.7^b^	5.52 ± 0.8
*Jania longifurca* Zanardini		36.3 ± 2.8^c^	28.2 ± 2.9^a^	1.33 ± 0.4^d^	46.2 ± 3.9^b^	35.8 ± 3.9^a^	5.64 ± 0.6^b^
*Corallina mediterranea* J.E. Areschoug		60.3 ± 6.1^b^	5.28 ± 1.5^d^	1.31 ± 0.2^d^	52.5 ± 5.2^a^	31.1 ± 3.8^b^	6.02 ± 0.8^a^
**Stanly station**							
*Codium decorticatum* (Woodward) M.A. Howe	Green	68.5 ± 8.4^a^	10.3 ± 3.8^c^	6.7 ± 3.7^a^	45.6 ± 5.5^b^	24.6 ± 3.8^c^	5.36 ± 0.9^b^
*Ulva fasciata* Delile		69.6 ± 9.9^a^	7.79 ± 2.9^d^	1.95 ± 0.3^d^	50.3 ± 6.2^a^	33.4 ± 9.5^b^	5.94 ± 0.2^a^
*Corallina officinalis* Linnaeus	Red	36.7 ± 4.8^c^	27.9 ± 9.9^a^	3.07 ± 0.7^c^	40.1 ± 4.1^b^	29.8 ± 4.5^c^	4.92 ± 1.2^b^
*Gracilaia dura* (C. Agardh) J. Agardh		54.3 ± 7.6^b^	11.3 ± 4.2^c^	2.94 ± 0.4^c^	51.7 ± 6.6^a^	32.9 ± 9.1^b^	6.09 ± 1.1^a^
**Eastern harbor**							
*Ulva fasciata* Delile	Green	76.5 ± 10.2^a^	11.6 ± 5.7^b^	2.87 ± 0.7^c^	50.0 ± 8.6^a^	33.2 ± 6.0^b^	5.94 ± 0.5^a^
*Ulva lactuca* Linnaeus		65.3 ± 8.7^a^	14.4 ± 7.8^b^	1.6 ± 0.08^d^	50.2 ± 8.9^a^	28.1 ± 5.8^c^	5.71 ± 0.3^a^
*Ulva linza* Linnaeus		75.5 ± 9.9^a^	6.67 ± 2.9^d^	3.79 ± 0.9^b^	50.9 ± 9.1^a^	27.6 ± 5.4^c^	5.84 ± 0.2^a^
*Corallina officinalis* Linnaeus	Red	35.3 ± 2.1^c^	15.4 ± 7.9^b^	1.98 ± 0.2	39.3 ± 6.5^b^	25.8 ± 5.0^c^	4.6 ± 0.4^b^
*Griffithsia opuntioides* J. Agardh		58.4 ± 7.6^b^	11.0 ± 5.0^c^	7.2 ± 6.2^a^	40.8 ± 8.0^b^	39.0 ± 6.9^a^	5.5 ± 0.5^a^
*Petalonia fascia* (O.F. Müller) C. Agardh	Brown	45.3 ± 5.6^c^	5.19 ± 2.2^d^	1.71 ± 0.4^d^	41.3 ± 7.9^b^	39.2 ± 7.0^a^	4.65 ± 0.8^a^
**El Mex Bay**							
*Cladophora glomerata* (Linnaeus) Kützing	Green	71.2 ± 9.3^a^	12.4 ± 6.7^c^	3.44 ± 0.4^b^	43.6 ± 5.6^b^	42.1 ± 4.1^a^	5.74 ± 0.4^a^
*Cladophora pellucida* (Hudson) Kützing		72.5 ± 8.9^a^	8.30 ± 4.7^d^	6.93 ± 0.7^a^	42.5 ± 5.0^b^	34.1 ± 3.6	5.47 ± 0.5^a^
*Ulva fasciata* Delile		78.0 ± 9.9^a^	9.90 ± 4.9^c^	3.85 ± 0.5^b^	50.1 ± 5.9^a^	40.4 ± 4.4^a^	6.28 ± 0.8^a^
*Ulva linza* Linnaeus		65.6 ± 6.9^a^	7.25 ± 4.5^d^	6.93 ± 0.6^a^	42.5 ± 5.1^b^	34.1 ± 3.4^b^	5.47 ± 0.5^a^
*Grateloupia doryphora* (Mont.) A. Howe	Red	70.4 ± 8.5^a^	7.30 ± 4.0^d^	4.37 ± 0.7^b^	51.6 ± 6.2^a^	27.2 ± 3.1^c^	5.91 ± 1.1^a^
*Griffithsia opuntioides* J. Agardh		42.3 ± 5.6^c^	11.2 ± 6.9^c^	1.45 ± 0.1^d^	34.6 ± 3.6^c^	24.7 ± 2.9^c^	4.16 ± 0.5^b^
*Corallina officinalis* Linnaeus		44.7 ± 5.9^c^	18.5 ± 9.7^b^	1.69 ± 0.2^d^	50.6 ± 5.6^a^	31.7 ± 3.1^b^	5.89 ± 1.2^a^
*Gracilaia dura* (C. Agardh) J. Agardh		35.6 ± 3.0^c^	7.80 ± 4.9^c^	2.51 ± 0.3	52.3 ± 5.8^a^	27.6 ± 2.9^c^	5.91 ± 0.9^a^
**El‐Dekheila coast**							
*Ulva linza* Linnaeus	Green	66.8 ± 6.9^a^	14.6 ± 4.9^b^	3.79 ± 0.5^b^	51.9 ± 9.1^a^	42.4 ± 8.4^a^	6.52 ± 0.5^a^
*Cladophora glomerata* (Linnaeus) Kützing		68.5 ± 7.9^a^	13.3 ± 4.2^b^	7.34 ± 2.8^a^	40.5 ± 8.5^b^	38.9 ± 6.9^a^	5.49 ± 0.6^a^
Max.		78.0 ± 9.9	28.2 ± 2.9	7.9 ± 1.7	52.5 ± 5.2	43.0 ± 2.1	6.52 ± 0.5
Min.		28.5 ± 1.4	3.25 ± 0.1	1.05 ± 0.03	34.6 ± 3.6	24.4 ± 0.6	4.14 ± 0.9

*Note:* ^a–e^Different superscript letters on the same column indicate a significant difference between stations at *p* ≤ 0.05 level of significance.

The total protein content ranged from 24.4% ± 0.6% DW in *P. tetrastromatica* to 43.0% ± 2.1% DW in *U. linza* from Abu‐Qir. This variation could be attributed to differences in chemical compositions, spatial variations, and distinct periods (El‐Sayed and Ismail 2022; Ismail et al. 2017). The caloric content can be determined on the basis of the concentrations of carbohydrates, protein, and lipids and is indicative of food quality. The seaweed samples examined had low caloric values, ranging from 2.47 ± 0.2 kcal/100 g DW (*G. opuntioides* from El Mex) to 4.85 ± 0.3 kcal/100 g DW ( *C. officinalis* from Sidi Bishr).

### 3.2 Pigment Contents

In the current work, *Cl*. *The glomeruli* from El Mex presented the greatest amount of photosynthetic pigments (chlorophyll *a*: 90.3 ± 13.1 mg/g FW; carotenoid: 20.9 ± 9.1 mg/g FW), whereas *Ulva linza* from Eastern Harbor presented the greatest carotenoid (24.6 ± 9.1 mg/g FW) and carotene (8.23 ± 0.3 mg/100 g DW) contents, as shown in Table 3. On the other hand, the brown seaweed *P. boryana* from Abu‐Qir presented the lowest pigment concentration (chlorophyll *a*: 0.39 ± 0.09; total chlorophyll: 3.08 ± 0.1 mg/g FW), whereas red species ( *Grateloupia doryphora* and *G. opuntioides*) from El Mex presented relatively high carotenoid (8.95 ± 0.6 mg/g FW) and *β*‐carotene (8.95 ± 0.6 mg/100 g DW) contents. The highest lycopene content was detected in *Cl. glomerata* collected from the El Dekheila coast (7.69 ± 0.8 mg/100 g DW). Differences in pigment content could have been due to the extraction techniques, algal species, and their classification (Garcia‐Perez et al. 2022; Ismail, El Zokm, and Miranda 2023). Green seaweeds such as *U. linza and Cl. glomerata* are known for their high protein and photosynthetic pigment contents and are used in human and animal feeds, nutritional products, food supplements, and cosmetics because of their antioxidant properties (Ismail, El Zokm, and Miranda 2023).

**TABLE 3:** Spatial variation in pigment content of the collected seaweed.

Allocation/Seaweed spp.	Group	Chl *a* (mg/g FW)	Total Chl (mg/g FW)	Carotenoid (mg/g FW)	β‐carotene (mg/100 g DW)	Lycopene (mg/100 g DW)
**Abu Qir Bay**						
*Dictyota dichotoma* (Hudson) Lamouroux	Brown	26.2 ± 0.8^c^	42.0 ± 10.0^c^	8.58 ± 0.8^c^	2.96 ± 0.04^d^	4.40 ± 0.7^b^
*Dictyota linearis* (C.Agardh) Greville		45.0 ± 2.5^b^	74.0 ± 4.0^b^	16.8 ± 1.8^b^	2.31 ± 0.03^d^	5.13 ± 0.9^a^
*Padina boryana* Thivy		1.39 ± 0.09^e^	3.08 ± 0.1^e^	0.83 ± 0.01^e^	0.50 ± 0.09^e^	0.89 ± 0.08^e^
*Padina pavonia* (Linnaeus) J.V. Lamouroux		20.8 ± 1.9^c^	51.8 ± 8.0^c^	8.02 ± 0.7^c^	2.99 ± 0.05^d^	3.45 ± 0.6^c^
*Padina tetrastromatica* Hauck		0.98 ± 0.08^e^	7.70 ± 1.0	0.58 ± 0.1^e^	0.55 ± 1.0^e^	3.94 ± 0.5^c^
*Codium decorticatum* (Woodward) M.A. Howe	Green	31.3 ± 0.7^c^	52.7 ± 7.0^c^	12.0 ± 0.7^b^	2.63 ± 0.09^d^	0.63 ± 0.04^e^
*Ulva fasciata* Delile		20.8 ± 0.9^c^	41.8 ± 6.0^c^	8.02 ± 0.3^c^	2.25 ± 0.03^d^	0.73 ± 0.06^e^
*Ulva lactuca* Linnaeus		14.2 ± 0.8^d^	44.0 ± 4.5^c^	3.53 ± 0.1^d^	2.12 ± 0.09^d^	6.85 ± 0.8^a^
*Ulva linza* Linnaeus		21.6 ± 0.5^c^	53.2 ± 3.2^c^	6.30 ± 0.5^c^	5.82 ± 0.3^b^	2.51 ± 0.09^d^
*Amphiroa rigida* J.V. Lamouroux	Red	7.14 ± 0.4^d^	19.3 ± 1.9^d^	1.90 ± 0.4^e^	1.23 ± 0.01^e^	1.17 ± 0.01^e^
*Chondracanthus acicularis* (Roth) Fredericq		9.14 ± 0.5^d^	26.8 ± 5.8^d^	8.83 ± 0.8^c^	4.36 ± 0.8^c^	3.00 ± 0.2^b^
*Corallina officinalis* Linnaeus		21.8 ± 0.6^c^	35.5 ± 1.2^d^	5.57 ± 0.09^d^	2.30 ± 0.06^d^	6.52 ± 0.5^a^
*Jania rubens* (Linnaeus) Lamouroux		25.9 ± 0.8^c^	39.6 ± 6.0^c^	10.8 ± 1.7^b^	2.23 ± 0.05^d^	1.93 ± 0.05^d^
*Jania longifurca* Zanardini		26.6 ± 0.6^c^	67.0 ± 3.0^b^	13.7 ± 1.9^b^	2.38 ± 0.09^d^	2.41 ± 0.08^d^
*Polysiphona elongata* (Hudson) Sprengel		5.71 ± 0.2^e^	35.5 ± 1.7^d^	5.52 ± 0.8^d^	1.16 ± 0.02^e^	0.07 ± 0.01^e^
*Pterocladia capillacea* (Gmelin) Bornet		22.7 ± 1.7^c^	48.1 ± 12.0^c^	10.9 ± 1.3^b^	3.19 ± 0.5^c^	1.09 ± 0.07^d^
**Sidi Bishr station**						
*Ulva fasciata* Delile	Green	19.5 ± 3.8^c^	45.9 ± 9.6^a^	5.05 ± 0.4^c^	1.95 ± 0.05^e^	3.56 ± 0.6^c^
*Ulva linza* Linnaeus		43.0 ± 4.7^b^	69.2 ± 23.0^b^	9.34 ± 0.9^b^	1.43 ± 0.03^e^	1.16 ± 0.04^e^
*Pterocladia capillacea* (Gmelin) Bornet	Red	6.32 ± 1.9^e^	59.4 ± 4.2^b^	3.44 ± 0.2^d^	2.81 ± 0.7^d^	0.83 ± 0.01^e^
*Amphiroa rigida* J.V. Lamouroux		11.6 ± 2.6^c^	46.2 ± 2.9^c^	4.01 ± 0.4^d^	3.09 ± 0.9^c^	1.06 ± 0.07^e^
*Jania rubens* (Linnaeus) Lamouroux		18.2 ± 3.1^c^	49.6 ± 27.0^c^	7.20 ± 0.8^c^	1.35 ± 0.04^e^	4.07 ± 0.5^b^
*Corallina officinalis* Linnaeus		5.48 ± 1.6^e^	20.4 ± 1.9^d^	3.38 ± 0.1^d^	1.18 ± 0.02^e^	2.90 ± 0.2^d^
**Gleem station**						
*Ulva fasciata* Delile	Green	21.0 ± 3.1^c^	43.1 ± 12.0^c^	8.14 ± 2.6^c^	1.16 ± 0.8^e^	0.28 ± 0.04^e^
*Padina boryana* Thivy	Brown	5.44 ± 0.5^e^	43.0 ± 7.2^c^	3.26 ± 0.9^d^	1.27 ± 0.9^e^	0.89 ± 0.09^e^
*Corallina officinalis* Linnaeus	Red	7.08 ± 0.8^d^	52.0 ± 8.2^b^	1.92 ± 0.08^e^	1.16 ± 0.7^e^	0.14 ± 0.01^e^
*Pterocladia capillacea* (Gmelin) Bornet		9.09 ± 0.9^d^	23.7 ± 4.8^d^	6.12 ± 0.03^d^	4.80 ± 1.3^c^	3.86 ± 0.9^c^
*Jania rubens* (Linnaeus) Lamouroux		26.7 ± 4.7^c^	41.4 ± 17.0^c^	10.0 ± 2.7^b^	6.51 ± 2.1^b^	3.41 ± 0.6^c^
*Amphiroa rigida* J.V. Lamouroux		10.9 ± 2.9^d^	61.0 ± 9.8^b^	2.40 ± 0.9^e^	1.18 ± 0.9^e^	2.14 ± 0.3^d^
*Corallina elongata* J. Ellis & Solander		23.1 ± 3.0^c^	55.2 ± 16.0^b^	10.58 ± 1.3^c^	8.49 ± 2.4^a^	3.53 ± 0.7^b^
*Jania longifurca* Zanardini		32.1 ± 4.1^b^	77.7 ± 27.3^b^	12.0 ± 2.9^b^	7.99 ± 1.9^b^	4.25 ± 0.8^b^
*Corallina mediterranea* J.E. Areschoug		8.50 ± 0.9^d^	62.4 ± 9.9^b^	2.31 ± 0.5^e^	0.81 ± 0.04^e^	0.23 ± 0.03^e^
**Stanly station**						
*Codium decorticatum* (Woodward) M.A. Howe	Green	11.1 ± 7.8^d^	41.2 ± 11.1^c^	8.00 ± 1.2^c^	6.04 ± 2.8^b^	3.81 ± 0.9^c^
*Ulva fasciata* Delile		27.6 ± 10.0^b^	67.2 ± 17.6^b^	11.4 ± 7.4^b^	1.21 ± 0.3^e^	0.8 ± 0.08^e^
*Corallina officinalis* Linnaeus	Red	10.0 ± 7.6^d^	44.7 ± 7.9^c^	2.04 ± 0.9^e^	1.18 ± 0.4^e^	0.9 ± 0.04^e^
*Gracilaia dura* (C.Agardh) J. Agardh		16.6 ± 9.8^c^	31.7 ± 12.3^d^	8.50 ± 1.7^c^	6.49 ± 2.4^b^	3.54 ± 0.8^c^
**Eastern harbor**						
*Ulva fasciata* Delile	Green	17.0 ± 7.0^c^	35.7 ± 11.5^d^	7.10 ± 0.8^c^	1.24 ± 0.4^e^	1.65 ± 0.8^d^
*Ulva lactuca* Linnaeus		25.1 ± 9.9^b^	49.6 ± 19.9^c^	4.75 ± 0.4^d^	1.14 ± 0.2^e^	0.5 ± 0.08^e^
*Ulva linza* Linnaeus		85.9 ± 11.9^a^	140.8 ± 22.1^a^	24.6 ± 9.0^a^	8.23 ± 0.3^a^	1.66 ± 0.7^d^
*Corallina officinalis* Linnaeus	Red	5.59 ± 0.6^e^	45.1 ± 9.9^c^	1.29 ± 0.2^e^	0.9 ± 0.06^e^	1.82 ± 0.7^d^
*Griffithsia opuntioides* J. Agardh		13.3 ± 6.1^d^	35.0 ± 14.6^d^	8.42 ± 2.2^c^	3.25 ± 0.8^d^	2.74 ± 0.9^c^
*Petalonia fascia* (O.F. Müller) C. Agardh	Brown	26.3 ± 9.9^c^	57.6 ± 12.3^b^	2.44 ± 0.3^e^	1.15 ± 0.3^e^	0.98 ± 0.5^d^
**El Mex Bay**						
*Cladophora glomerata* (Linnaeus) Kützing	Green	90.3 ± 13.1^a^	150.1 ± 22.9^a^	20.9 ± 9.1^a^	5.81 ± 0.05^b^	3.57 ± 0.6^c^
*Cladophora pellucida* (Hudson) Kützing		81.3 ± 10.9^a^	142.9 ± 19.9^a^	19.6 ± 8.1^a^	3.44 ± 0.3^d^	6.22 ± 0.8^a^
*Ulva fasciata* Delile		12.1 ± 7.8^d^	71.3 ± 10.0^b^	3.6 ± 0.8^e^	1.66 ± 0.06^e^	1.77 ± 0.8^d^
*Ulva linza* Linnaeus		18.4 ± 8.9^c^	79.7 ± 10.9^b^	2.90 ± 0.8^e^	1.55 ± 0.4^e^	3.25 ± 0.4^c^
*Grateloupia doryphora* (Mont.) A. Howe	Red	3.89 ± 0.8^e^	12.0 ± 0.6.9^e^	1.1 ± 0.01^e^	0.95 ± 0.6^e^	7.44 ± 0.9^a^
*Griffithsia opuntioides* J. Agardh		20.1 ± 3.1^c^	53.8 ± 15.9^c^	8.78 ± 0.9^c^	2.8 ± 0.1^d^	0.3 ± 0.03^e^
*Corallina officinalis* Linnaeus		8.16 ± 1.0^d^	25.1 ± 5.8^d^	1.3 ± 0.09^e^	1.18 ± 0.5^e^	2.90 ± 0.4^c^
*Gracilaia dura* (C. Agardh) J. Agardh		7.00 ± 0.9^d^	21.5 ± 4.9^d^	1.25 ± 0.05^e^	0.15 ± 0.8^e^	4.35 ± 0.7^d^
**El‐Dekheila coast**						
*Ulva linza* Linnaeus	Green	10.1 ± 2.1^d^	31.1 ± 6.7^d^	7.4 ± 0.05^c^	5.3 ± 1.9^d^	6.72 ± 0.5^a^
*Cladophora glomerata* (Linnaeus) Kützing		18.2 ± 3.2^c^	55.9 ± 10.1^c^	0.7 ± 0.08^e^	4.60 ± 0.8^d^	7.69 ± 0.8^a^
Max.		90.3 ± 13.1	150.1 ± 22.9	24.6 ± 9.1	8.23 ± 0.3	7.69 ± 0.8
Min.		0.98 ± 0.08	3.08 ± 0.1	0.7 ± 0.08	0.15 ± 0.8	0.07 ± 0.01

*Note:* ^a–e^Different superscript letters on the same column indicate a significant difference between stations at the *p* ≤ 0.05 level of significance.

### 3.3 Accessory Pigment Contents

**FIGURE 2:**
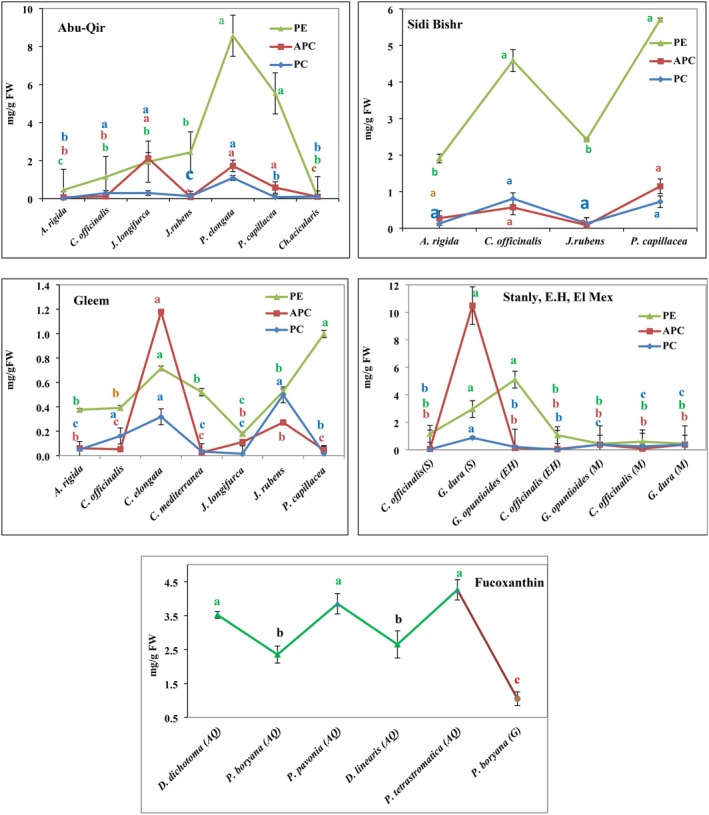
The special pigments levels of the red (PE, APC & PC) and brown tested species (Fuc.). Similar superscript letters indicate insignificant difference between species at *p* ≤ 0.05 level of significance.

Figure 2 depicts the phycobiliprotein pigments (phycoerythrin [PE], allophycocyanine [APC], and phycocyanin [PC]) of red species, as well as the fucoxanthin of the selected brown seaweeds. This figure shows a significant difference (*p* < 0.05) between the red species that were evaluated. *P. elongata* had the highest PE and PC values (8.56 ± 0.9 and 1.09 ± 0.05 mg/g FW, respectively) in the Abu‐Qir region, whereas *J. longifurca* samples from the same area presented the maximum APC concentration (2.12 ± 0.15 mg/g FW). Additionally, *P. capillacea* from Sidi Bishr presented significant amounts of PEs and APCs (5.71 ± 0.51 and 1.15 ± 0.07 mg/g FW, respectively), whereas *C. officinalis* presented the highest concentration of PCs (0.808 ± 0.29 mg/g FW).

In another region, such as the Gleem coast, *P. capillacea* was found to have a high PE composition (1 ± 0.06 mg/g FW), *C. elongata* featured a high APC content (1.18 ± 0.08 mg/g FW), and *J. rubens* presented the highest PC value (0.5 ± 0.04 mg/g FW). Notably, high levels of phycobiliproteins were detected in *G. dura* (in Stanly) and *G. opuntioides* (in E.H.) (PE: 2.96 ± 0.29, APC: 10.49 ± 1.20, and PC: 0.87 ± 0.19 mg/g FW; PE: 5.10 ± 0.69, APC: 0.13 ± 0.03, and PC: 0.21 ± 0.09 mg/g FW). Similarly, in the El Mex research area, those species ( *G. dura* and *G. opuntioides*) presented high and identical PE (0.44 ± 0.17 mg/g), APC (0.38 ± 0.12 mg/g), and PC (0.38 ± 0.19 mg/g FW) values, whereas *C. officinalis* presented significantly polar PE values of 0.59 ± 0.19 mg/g FW at that location.

In contrast, high amounts of the fucoxanthin compound (4.26 ± 0.84, 3.85 ± 0.59, and 3.52 ± 0.51 mg/g FW) were detected in *P. tetrastromatica, P. pavonia*, and *D. dichotoma*, respectively, from Abu‐Qir (Figure 2). The levels of fucoxanthin in *P. boryana* from Gleem and *G. doryphora* from El Max were 1.05 ± 0.60 and 0.89 ± 0.27 mg/g FW, respectively. This discrepancy could be caused by biological criteria such as the algal tissue type, seaweed species, thickness, thallus shape, and configuration of the photosynthesis system, as well as physicochemical factors (light, density, sea depth, photoperiod, temperature, salinity, pH, and nutrient salts) (El‐Sayed and Ismail 2022; Ismail and Osman 2016; Wu 2016). The environmental factors for our work are temperature (21.09°C), salinity = 38‰, pH = 8.0. According to the current work, *C. officinalis* is the most prevalent species that carries phycobiliprotein pigments throughout all the study locations. Additionally, *P. capillacea* has high PE levels in Gleem and Sidi Bishr. On the other hand, *P. boryana* containing fucoxanthin pigments was found in Gleem and Abu Qir. This could be explained by morphological traits, the extraction technique, and the surrounding conditions (Ismail et al. 2016; McDonnell et al. 2024).

### 3.4 Vitamin Contents

**FIGURE 3:**
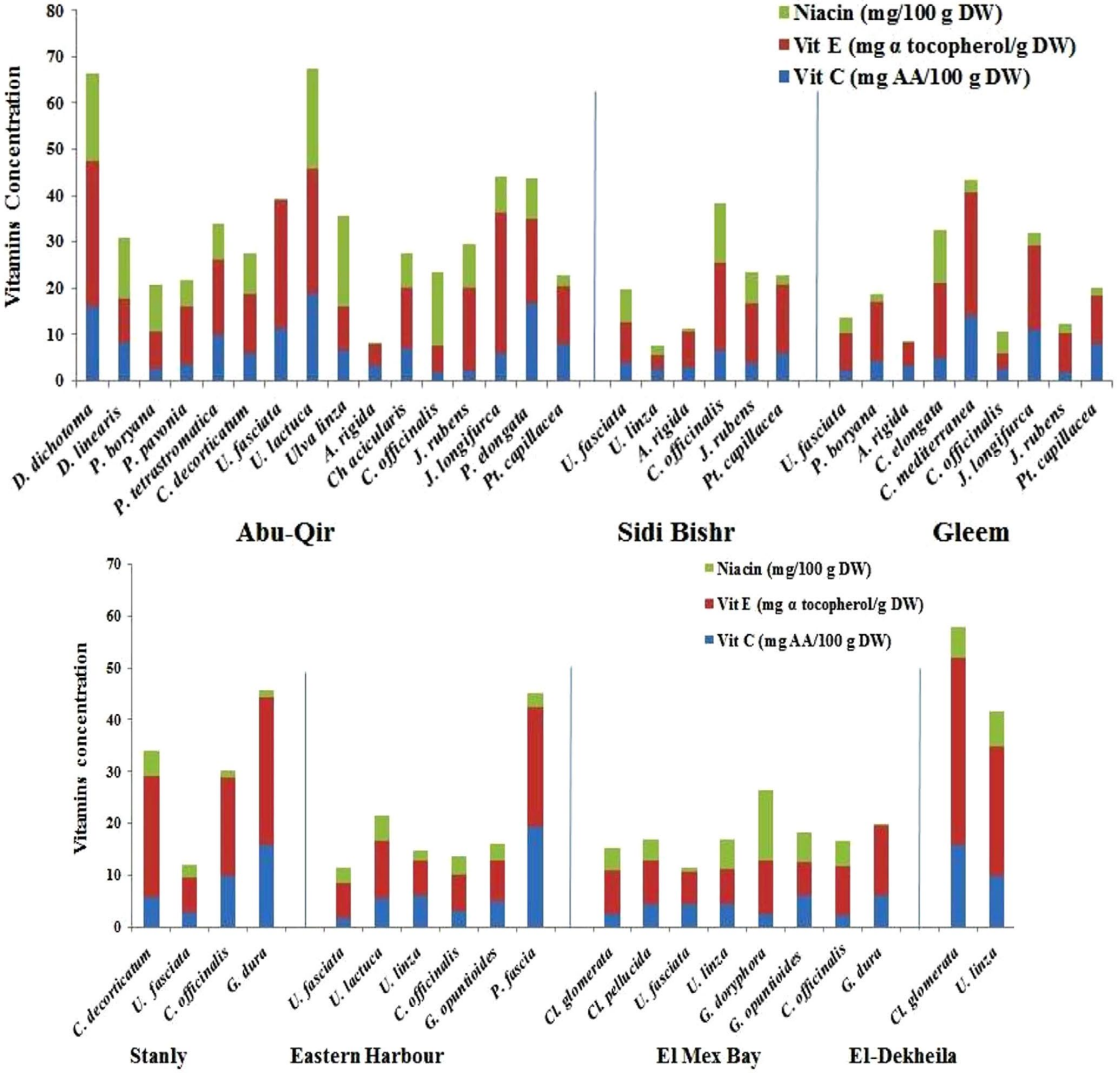
Variation in vitamins content of the collected species.

Figure 3 shows that, compared with the other seaweed samples studied, the brown alga *P. fascia* had the highest vitamin C value (19.2 ± 8.6 mg AA/g DW), whereas *U. fasciata* had the lowest value (1.66 ± 0.1 mg AA/g DW) in the same region (E.H.). However, *Cl*. *The glomeruli* from the El Dekheila coast presented the highest vitamin E concentration (35.9 ± 5.2 mg α tocopherol/g DW), whereas *U. linza* (Sidi Bishr) presented the lowest (3.17 ± 0.4 mg α tocopherol/g DW). Niacin (vitamin B3) levels were higher in *U. lactuca* (21.8 ± 2.1 mg/100 g DW in Abu‐Qir) than in the other types, but a lower value was estimated for *A. rigida* from the Gleem station (0.20 ± 0.03 mg/100 g DW). Several characteristics, such as seaweed type, developmental phase, geographic site, salinity level, period, light, and temperature, influence differences in vitamin concentrations (Ismail, El Zokm, and Miranda 2023).

### 3.5 Fatty Acid Composition

The fatty acid profiles and their compositions were determined in three common algal species (*P. boryana, P. capillacea, and U. fasciata*) at most stations and were identified on the basis of their nutritional value as a percentage of total fatty acids (FAs) and are summarized in Table 4. These species are characterized by high abundance and biomass and have been documented as edible and healthy species (Ismail et al. 2017; El Zokm et al. 2021). Among the three seaweed samples, 12 FA compounds were found. The total fatty acid ratio (∑FA) ranged from 96.91% ± 4.98% in *P. boryana* to 98.16% ± 4.06% in *U. fasciata* , reflecting the diversity in FA compounds for each species. Table 4 shows that the range of saturated fatty acids (SFAs) recorded was 78.59% ± 3.85% of the total FAs in *P. boryana* to 88.02% ± 3.53% in *U. fasciata*, whereas the content of monounsaturated fatty acids (MUFAs) varied from 5.37% ± 0.38% of the total FAs in *P. capillacea* to 18.07% ± 0.8% in *P. boryana* . The percentage of polyunsaturated fatty acids (PUFAs) ranged from 0.47% ± 0.04% of the total FAs in *P. boryana* to 5.26% ± 0.74% of the total FAs in *P. capillacea* . Thus, the abundance of SFAs and unsaturated fatty acids for each species was in the order of SFAs > MUFAs > PUFAs. Compared with the other two species, *U. fasciata* has a high SFA content of 88.02% ± 3.53%. Palmitic and stearic acids were the major SFAs in all species, which is in agreement with the findings of previous studies (El Baz et al. 2013; Moustafa and Batran 2014; Ismail 2017), with maximums of 75.48% ± 3.01% and 7.44% ± 0.29%, respectively, in *U. fasciata* , followed by myristic acid, which was much more abundant in *P. boryana* than in the other species, with values of 6.6% ± 0.36%. Furthermore, only *P. boryana* has eicosanoic SFAs, which have a concentration of 0.58% ± 0.03% and operate as a cellular process bioregulator (De Alencar et al. 2018).

**TABLE 4:** The fatty acids (FAs) content of the seaweed species (% of total of fatty acid).

Fatty acids (FAs)	Brown	Red	Green
	*P. boryana*	*P. capillacea*	*U. fasciata*
Decanoic acid (C10:0)	ND	0.58 ± 0.04	0.38 ± 0.02
Dodecanoic acid (C12:0)	0.76 ± 0.04	4.84 ± 0.25	1.26 ± 0.07
Myristic acid (C14:0)	6.6 ± 0.36	5.19 ± 0.33	2.67 ± 0.09
Pentadecanoic acid (C15:0)	0.58 ± 0.03	0.86 ± 0.06	0.79 ± 0.05
Palmitic acid (C16:0)	67.72 ± 2.94	70.62 ± 2.98	75.48 ± 3.01
Stearic acid (C18:0)	2.35 ± 0.10	4.19 ± 0.20	7.44 ± 0.29
Eicosanoic acid (C20:0)	0.58 ± 0.03	ND	ND
Palmitoleic acid (C16:1) (*n‐*7)	4.84 ± 0.35	0.54 ± 0.03	1.55 ± 0.08
Oleic acid (C18:1) (*n‐*9)	13.23 ± 0.45	4.83 ± 0.35	8.11 ± 0.39
Linoleic acid (C18:2) (*n‐*6)	0.47 ± 0.04	0.40 ± 0.03	0.48 ± 0.04
Eicosatetraenoic acid (C20:4) (ARA) (*n‐*6)	ND	0.86 ± 0.06	ND
Eicosapentaenoic acid (EPA) (C20:5) (*n‐*3)	ND	4.00 ± 0.65	ND
∑ SFA	78.59 ± 3.85	86.28 ± 3.86	88.02 ± 3.53
∑ MUFA	18.07 ± 0.80	5.37 ± 0.38	9.66 ± 0.49
∑ PUFA	0.47 ± 0.04	5.26 ± 0.74	0.48 ± 0.04
∑ FA	97.13 ± 4.69	96.91 ± 4.98	98.16 ± 4.06

*Note:* The results are expressed as (mean ± SD) of the triplicate samples (*n* = 3); ND: not detected. ∑ FA, ∑SFA, ∑MUFA, ∑PUFA: The sum of fatty acids (FA) concentrations for each species, saturated fatty acids; monounsaturated fatty acids; and polyunsaturated fatty acids; respectively. N: diversity in FA compounds for each species. (ARA): Arachidonic acid (common name).

Table 4 shows that *P. boryana* had the highest proportion of MUFAs (18.07% ± 0.8%); in particular, oleic acid (13.23% ± 0.45%) was more prevalent in this species than in other species, which is consistent with oleic acid being one of the key MUFAs in brown seaweed (Engelen et al. 2018; Vizetto‐Duarte et al. 2015). Furthermore, the highest level of PUFA (5.26% ± 0.74%) was found only in red seaweed ( *P. capillacea* ); this refers to the presence of linoleic PUFA (*n‐*6), with a value of 0.47% ± 0.04%. In addition, only this species has the maximum abundance of eicosatetraenoic acid (0.86% ± 0.06%) and eicosapentaenoic acid (4.00% ± 0.65%), in accordance with Shaltout and El‐Din (2015) and De Alencar et al. (2018). Notably, linoleic acid was the predominant *n‐*6 PUFA in all the tested species in close proportions. Therefore, this variation in content may be related to environmental conditions, such as the temperature of the surrounding water, extraction method, genetic disparity, drying method of the seaweed, morphology of each species, and seasonal distinctions (Ismail et al. 2016; McDonnell et al. 2024). The World Health Organization and the Food and Agriculture Organization (WHO/FAO Expert Consultation 2003) recommend reducing the consumption of saturated fatty acids (SFAs) to less than 10% of total energy intake. Unsaturated fatty acids replace saturated with unsaturated fatty acids, especially polyunsaturated fatty acids (PUFAs). Basically, the WHO/FAO Expert Consultation 2003 recommend that adults and children follow a diet where total fatty acid intake is moderate, focusing on replacing harmful saturated fatty acids with beneficial unsaturated fatty acids to promote overall health and prevent chronic diseases. On the other hand, the European Food Safety Authority (EFSA 2010) recommends that adults consume 20%–35% of their total energy from fatty acids and limit saturated fatty acids (SFAs). They also recommend consuming 250 mg of EPA daily. The results revealed that *U. fasciata* contains high amounts of total fatty acids (∑FAs) and SFA, particularly palmitic acid, which aligns with previous findings (Ismail 2017). This finding points to the green marine alga ( *U. fasciata* ) as a basis for biofuel production owing to the long straight chain of SFAs, resulting in a high‐quality standard and a quick ignition period (Moustafa and Batran 2014; Shaltout and El‐Din 2015). On the other hand, the tested red seaweed ( *P. capillacea* ) is a good source of nutrients for human health, which is consistent with the findings of De Alencar et al. (2018) and Rocha et al. (2021). Likewise, *P. boryana* species have applications in medical fields because of their high ratios of *n‐*7 and *n‐*9 MUFAs, as they exhibit strong antimicrobial activity and aid in resistance to several pathogenic diseases (Moustafa and Batran 2014).

### 3.6 Nutritional Values

Table 5 shows the nutritional values of the three seaweed types tested: *P. boryana, P. capillacea*, and *U. fasciata* . The index ratio of PUFAs/SFAs reflects the nutritional value of foods such as seaweed and their impact on cardiovascular health, suggesting that all PUFAs can reduce harmful cholesterol (LDL‐cholesterol) and blood cholesterol levels, whereas all SFAs contribute to elevated cholesterol levels. Therefore, the positive effects increase with increasing percentage (Chen and Liu 2020). Therefore, as shown in Table 4, *P. capillacea clearly* has a higher ratio of PUFAs/SFAs (0.06 ± 0.01) than the other tested species and is higher than that reported in a previous study by De Alencar et al. (2018), which referred to the tested *P. capillacea* as the sole species that presented the maximum concentration of PUFAs. This finding indicates that the red alga ( *P. capillacea* ) used in the present work has positive effects on heart health and nutritional value (Chen and Liu 2020; De Alencar et al. 2018; Rocha et al. 2021).

**TABLE 5:** The nutritional indices for the tested seaweed species.

Nutritional indices	Brown	Red	Green
	*P. boryana*	*P. capillacea*	*U. fasciata*
∑PUFAs/ ∑SFAs	0.06 ± 0.01	0.06 ± 0.01	0.05 ± 0.01
∑ ω3	ND	4.00 ± 0.65	ND
∑ ω6	0.47 ± 0.04	1.26 ± 0.09	0.48 ± 0.04
∑ ω6/∑ω3	0.47 ± 0.04	0.32 ± 0.13	0.48 ± 0.04
UI	19.01 ± 0.88	29.61 ± 3.28	10.62 ± 0.57
AI	5.12 ± 0.42	9.05 ± 0.56	8.62 ± 0.49
TI	8.30 ± 3.34	4.33 ± 0.28	16.88 ± 3.39
H/H	0.18 ± 0.04	0.13 ± 0.06	0.11 ± 0.04

Abbreviations: AI, Atherogenic index; H/H, Hypocholesterolemic/Hypercholesterolemic index; TI, Thrombogenic index; UI, Unsaturation index.

Conversely, the ∑*n‐*6/∑*n‐*3 ratios for *P. boryana, P. capillacea*, and *U. fasciata* were 0.47 ± 0.04, 0.32 ± 0.13, and 0.48 ± 0.04, respectively. These values are below the WHO and European Nutritional Societies (ENS) recommended thresholds of 10 and 5, respectively (Alles et al. 2014; Sánchez‐Machado et al. 2004). Compared with ENS, *P. capillacea* has a favorable *n‐*6/*n‐*3 ratio, which supports the use of *P. capillacea* for dietary purposes and for the prevention of numerous chronic diseases (Alles et al. 2014; Ismail 2017). One of the most intriguing results obtained in this study was that *P. capillacea* presented a higher unsaturation index (UI) value (29.61 ± 3.28) than the other species did. This finding indicates that this type is a rich source of PUFAs, which are critical for proper nutrition and health (Chen and Liu 2020; De Alencar et al. 2018; Rocha et al. 2021). The atherogenic index (AI), thrombogenic index (TI) and Hypocholesterolemic/hypercholesterolemic index (H/H) are associated with atherosclerosis, platelet aggregation, and cholesterol metabolism (Habeebullah et al. 2023). These indices are related to human health and the nutritional quality of seaweed (Szpunar‐Krok and Wondołowska‐Grabowska 2022). A low level of AI is present in *P. boryana* (5.12 ± 0.42), which is associated with a high percentage of MUFAs in these species, which contain different amounts of antioxidants, micronutrients, and phytochemicals, potentially positively influencing the formation of atherosclerosis (Ismail, El Zokm, and Miranda 2023; Ros 2003). On the other hand, *P. capillacea* had the lowest TI (4.33 ± 0.28), which may be attributed to its high levels of *n‐*3 PUFAs, which reduce blood clotting (El‐Beltagi et al. 2022). It also contains negatively charged sulfated polysaccharides that bind to platelet receptors through electrostatic interactions, thereby reducing the risk of clotting (Nagahawatta et al. 2023). A relatively high H/H ratio has a relatively low effect on cholesterol levels (Soares et al. 2021). The H/H value of our results was high in *P. boryana* (0.18 ± 0.04), which was related to the presence of fucoidan polysaccharides, which have been demonstrated to lower blood cholesterol and mitigate metabolic syndrome (El‐Beltagi et al. 2022). Moreover, *P. boryana* contains high concentrations of oleic *n‐*9 MUFAs, which lower the levels of LDL cholesterol and total cholesterol in the blood (Zhou et al. 2022).

On the basis of the results of the nutritional indicators, selecting a suitable type of seaweed, such as *P. capillacea* or *P. boryana* , may be a promising way to improve food quality in terms of its vital features, such as preventing neurological system disorders, heart diseases, and inflammation. These indicators also indicate that these seaweeds provide a suitable source of good fat for vegetarian people, who rival some types of fish (Soares et al. 2021).

### 3.7 Amino Acid Composition

**FIGURE 4:**
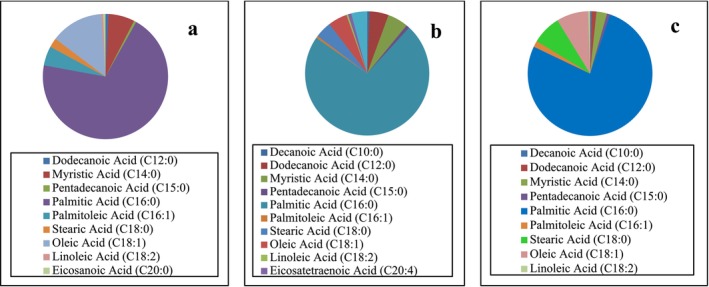
The fatty acids (%) content (a) *P. boryana*, (b) *P. capillacea*, and (c) *U. fasciata.*

**FIGURE 5:**
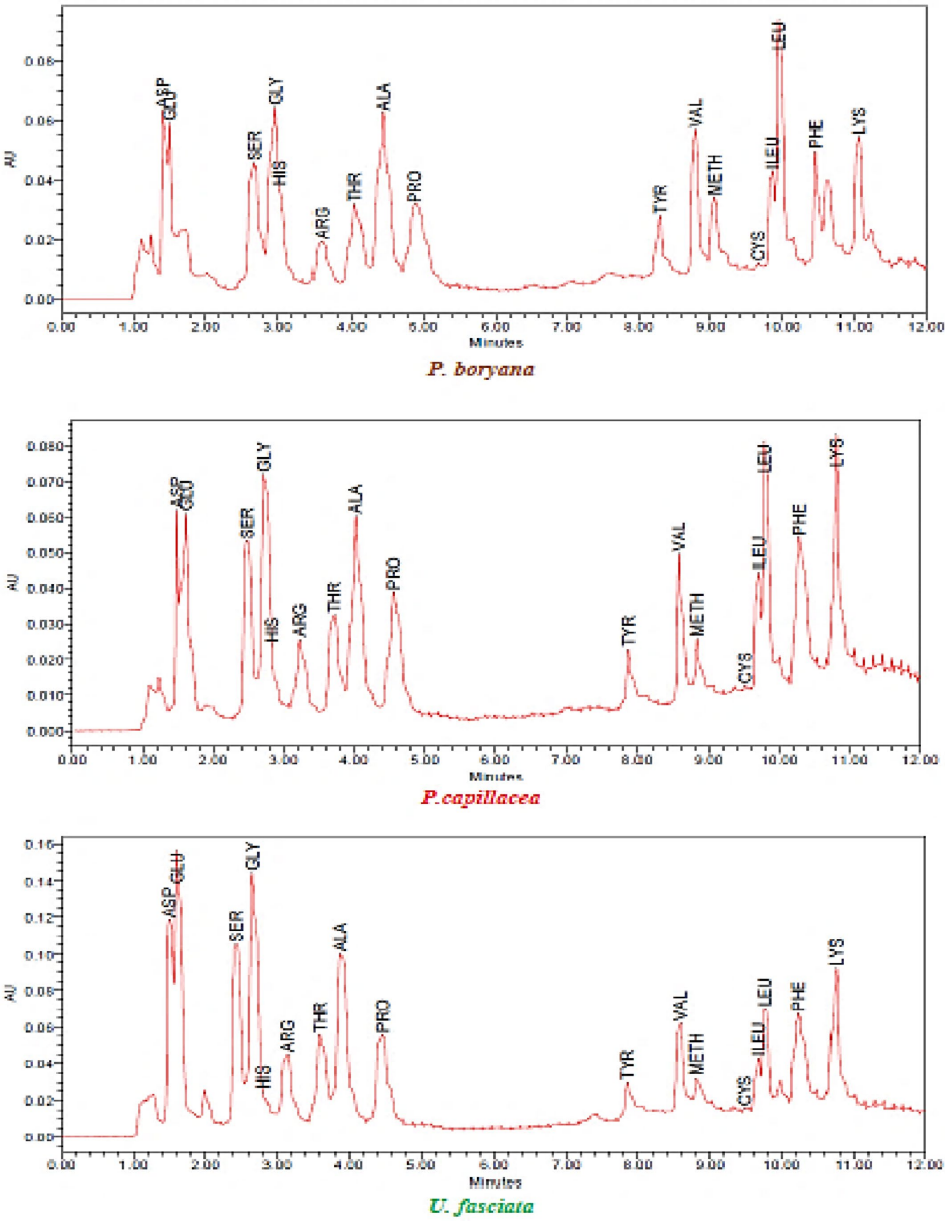
The amino acids of three seaweeds species *P. boryana, P. capillacea* and *U. fasciata*.

The contents and structures of amino acids from the selected three species (*P. boryana, P. capillacea* and *U. fasciata* ) at most locations are shown in Figure 4 and Table 6. The three studied species of marine seaweeds contained 17 amino acids (AAs). The total amino acid (TAA) content refers to the actual protein content in algae (Machado et al. 2020). Compared with the other species, *P. capillacea* presented the highest total amino acid (TAA) content (952.13 ± 16.70 mg/g). This could be due to variations in the chemical constitution, season of harvest, geographical position, and various environmental variables of the seaweed (Machado et al. 2020). Additionally, Fleurence et al. (2012) and Wan et al. (2019) reported that red seaweeds have the highest protein content, followed by green seaweed and brown ones. In the present work, brown seaweed had a high proportion of essential amino acids (EAAs) (374.20 ± 3.38 mg/g, or 39.47% ± 0.26% of total amino acids), which is in line with findings from previous works (Mohammed et al. 2021; Thiviya et al. 2022). The second‐ranked seaweeds were red seaweed (366.66 ± 6.83 mg/g, representing 38.51% ± 0.41% of the TAA), followed by green species (235.71 ± 4.05 mg/g, providing 24.84% ± 0.24% of the TAA). This is attributed to the higher levels of leucine, valine, and isoleucine (73.25 ± 0.28, 55.22 ± 0.23, and 53.26 ± 0.20 mg/g, respectively) of TAA in *P. boryana* than in the other species. Moreover, this may be related to the species type, algal location, seasonal fluctuations, preservation technique, and extraction process (Thiviya et al. 2022). The most prevalent essential amino acid in *P. capillacea* is phenylalanine (100.09 ± 2.50 mg/g, 10.51% ± 0.15%), suggesting that red seaweeds often have greater protein contents, particularly phenylalanine, and environmental parameters (Thiviya et al. 2022). On the other hand, high levels of nonessential amino acids (NEAAs) were recorded in *U. fasciata* (713.43 ± 12.53 mg/g, or 75.16% ± 0.76% TAA), like other types, because of the relatively high concentrations of aspartic and glutamic acids in this species (164.19 ± 2.16 and 336.95 ± 3.40 mg/g, respectively) of TAA. In addition, the morphological characteristics of *Ulva*, its age, the lifestyle of the algae, and external factors are important (Moustafa and Eladel 2015). Table 6 clearly shows that the alanine content was high in *P. boryana* (63.03 ± 3.60 mg/g, or 6.65% ± 0.27%), which could be related to several variables, such as season, algal size, and biological functions (Machado et al. 2020; Nagahisa et al. 1995). Moreover, the needs for essential amino acids (EAA) can vary individually based on factors such as age, sex, activity level, and overall health. Table S30 shows that the general requirements for adults for essential amino acids (EAA) mg/kg/day and its comparison with FAO/WHO/UNU (2007). The histidine, threonine, and methionine intake for adults from brown seaweeds ( *P. boryana* ) is 16.78, 16.39, and 16.85 mg/kg/day, respectively, which exceeds the adult daily recommendation by FAO/WHO/UNU (2007) (10, 15, and 15 mg/kg/day, respectively). On the other hand, the need for adults for isoleucine from *P. boryana* and *P. capillacea* is 22.83 and 21.16 mg/kg/day, respectively, which surpasses the adult daily of FAO/WHO/UNU (2007) (20 mg/kg/day). Additionally, the phenylalanine requirement from *P. capillacea* and *U. fasciata* is (42.90, and 27.14 mg/kg/day, respectively), which is an overdose adult daily of FAO et al. (2007) (25 mg/kg/day). Furthermore, the EAA/NEAA ratios were 0.65 ± 0.35 and 0.63 ± 0.69, respectively, in *P. boryana* and *P. capillacea* , indicating higher protein quality, better nutritional efficiency, and a superior chemical composition (Ismail 2017; Moustafa and Eladel 2015). A comparison of the EAA/NEAA ratios for *P. boryana* and *P. capillacea* with the ratios for soybeans (0.60) and white fish (0.59) revealed that brown and red species have amino acid compositions that surpass those of soybeans and white fish (Moustafa and Eladel 2015). The AAS (amino acid score) and EAAI (essential amino acid index) values for each tested sample are shown in Table 6 and were used to assess the quality of the protein. These values are based on the FAO/WHO/UNU (2007) recommended pattern of amino acids for adults as shown in Table 6 (Machado et al. 2020). The key limiting amino acids in *P. boryana, P. capillacea*, and *U. fasciata* were lysine, valine, and leucine, respectively, with the lowest AAS values (62.20% ± 0.44%, 83.31% ± 0.59%, and 41.00% ± 0.29%, respectively); therefore, the AAS indices for these specific seaweeds' proteins are 62.20, 83.31, and 41.00, respectively. Conversely, all species had threonine and methionine contents above the FAO/WHO/UNU standard (2007), which meant that the associated AAS values were greater than 100% (166.3%, and 245.8%) in *P. boryana* , (128.9%, and 178.0%) in *P. capillacea* , and (111.4%, and 124.4%) in *U. fasciata* , respectively; this indicated that the protein provides threonine and methionine essential amino acids in amounts proportional to the reference pattern of FAO/WHO/UNU. Also, threonine and methionine are indispensable for maintaining overall health. They participate in fundamental processes like protein synthesis, metabolism, immune function, and the building of structural components in the body (Machado et al. 2020). These variations could be caused by the species, reference protein, harvest season, location, physiological features of each species, and environmental factors (Machado et al. 2020). *P. boryana, P. capillacea*, and *U. fasciata* had EAAI values of 154% ± 0.95%, 122% ± 0.86%, and 82% ± 0.84%, respectively. The protein quality of Phaeophyta and Rhodophyta surpassed that of Chlorophyta; consequently, their amino acid profiles were more similar to those of the reference protein. As a result, these algae can be added to food products to improve their amino acid patterns and be employed as a great source of protein (Machado et al. 2020). A recent study suggested that *P. boryana* and *P. capillacea* may be sources of dietary proteins for feeding humans and animals and that their amino acid contents are of nutritional importance (Ismail 2017; Thiviya et al. 2022) (Figure 5).

**TABLE 6:** Amino acids (AAs) content (mg/g protein), amino acid score (AAS) and essential amino acid index (EAAI) for the seaweed species.

Amino acids (AAs)	FAO/WHO/UNU (2007) AAs scoring pattern (mg/g protein)	Brown	Red	Green
		*P. boryana*	*P. capillacea*	*U. fasciata*
**EAAs**				
Histidine	15	39.15 ± 0.14	17.49 ± 0.25	12.57 ± 0.07
Threonine	23	38.25 ± 0.10	29.64 ± 0.33	25.63 ± 0.09
Valine	39	55.22 ± 0.23	32.49 ± 0.06	32.65 ± 0.05
Methionine	16	39.32 ± 1.9	28.48 ± 2.98	19.90 ± 3.01
Isoleucine	30	53.26 ± 0.20	49.38 ± 0.18	25.83 ± 0.19
Leucine	59	73.25 ± 0.28	60.33 ± 0.28	24.20 ± 0.15
Phenylalanine	—	47.76 ± 0.37	100.09 ± 2.50	63.33 ± 0.30
Lysine	45	27.99 ± 0.16	48.76 ± 0.25	31.60 ± 0.19
∑ (EAAs)		374.20 ± 3.38	366.66 ± 6.83	235.71 ± 4.05
**NEAA**				
Aspartic		151.73 ± 1.80	88.63 ± 1.86	164.19 ± 2.16
Glutamic		158.39 ± 2.09	284.32 ± 3.38	336.95 ± 3.40
Serine		49.86 ± 0.24	39.58 ± 0.54	39.37 ± 0.15
Glycine		31.50 ± 0.69	33.94 ± 3.34	35.89 ± 0.56
Cysteine		4.07 ± 0.07	3.32 ± 0.03	2.64 ± 0.25
Arginine		36.14 ± 0.94	31.73 ± 0.15	33.98 ± 0.98
Alanine		63.03 ± 3.60	49.00 ± 0.28	47.22 ± 4.71
Proline		39.21 ± 0.19	27.47 ± 0.16	24.34 ± 0.10
Tyrosine		40.01 ± 0.22	27.48 ± 0.13	28.49 ± 0.18
∑ (NEAAs)		573.94 ± 9.84	585.47 ± 9.87	713.43 ± 12.53
TAAs		948.14 ± 13.22	952.13 ± 16.70	948.78 ± 16.58
% EAA		39.47 ± 0.26	38.51 ± 0.41	24.84 ± 0.24
% NEAA		60.53 ± 0.74	61.49 ± 0.59	75.16 ± 0.76
(EAAs)/(NEAAs)		0.65 ± 0.35	0.63 ± 0.69	0.33 ± 0.32
LAA		Lysine	Valine	Leucine
AAS (%)		62.20 ± 0.44	83.31 ± 0.59	41.00 ± 0.29
EAAI (%)		154 ± 0.95	122 ± 0.86	82 ± 0.83

*Note:* Values are means of three replicates (*n* = 3) ± standard deviations SD.

Abbreviations: AAS (%), Amino Acid Score; EAAI (%), essential amino acid index; EAAs, essential amino acids; LAA, limiting amino acid; NEAAs, non‐essential amino acids; TAAs, total amino acids.

## 4 Conclusion

Owing to their valuable bioactive content and nutritional value, seaweeds are considered promising biota for sustainable development. Understanding the composition of seaweeds is crucial at both the research and application levels because seaweeds provide a renewable source of energy and active materials for medical and nutritional applications, offering an effective and renewable solution for food and fuel shortages. This study is the first to evaluate more than 15 bioactive compounds in 51 marine seaweeds collected from seven different stations along the Alexandria coast, Egypt, over the same period. Few studies have been conducted globally on this topic, making this research groundbreaking. This study can serve as a foundation for further monitoring research and a better understanding of the most valuable species and areas.

On the basis of the data and valuable knowledge from the present study, seaweeds such as Chlorophyta *U. linza, Cl. Glomerata*, and *U. fasciata* are rich in vitamins (E and niacin), pigments, and total fatty acids, making them useful in human and animal feeds, food additives, cosmetics, and biofuel production. Some types of Rhodophyta have low fat and calorie contents, high carbohydrate contents, and high vitamin C contents, which can reduce the risk of obesity. The Rhodophyta sample *P. capillacea* is beneficial for human health because of its ARA and EPA PUFA contents. The brown alga *P. boryana* can be used in medical applications due to its high *n‐*7 and *n‐*9 MUFAs, strong antimicrobial activity, and disease resistance. The nutritional indicators of *P. capillacea* and *P. boryana* provide suitable vegetarian fat. Additionally, these species may be dietary protein sources for humans and animals, and their amino acid contents are significant. Future studies are needed to explore these viable economic resources and make large‐scale algae‐based food products commercially available.

## Author Contributions

**Mona M. Ismail:** conceptualization (equal), data curation (equal), formal analysis (equal), investigation (equal), methodology (equal), resources (equal), writing – original draft (equal), writing – review and editing (equal). **José M. Miranda Lopez:** formal analysis (equal), methodology (equal), validation (equal), visualization (equal), writing – review and editing (equal). **Abeer A. M. El‐Sayed:** conceptualization (equal), data curation (equal), formal analysis (equal), methodology (lead), writing – original draft (equal), writing – review and editing (equal).

## Ethics Statement

This study does not involve any human or animal testing.

## Conflicts of Interest

The authors declare no conflicts of interest.

## Supporting information


Data S1.

## Data Availability

The corresponding author will share the data underlying this article at a reasonable request.
