# Supplementary material for: Evaluation of the Spatial Variations in the Biochemical Composition of Seaweed Species Along the Coast of Alexandria, With a Focus on Fatty Acids and Total Amino Acids of the Prevalent Edible Species
Source: Food Sci Nutr. 2025 Jul 16;13(7):e70645. doi: 10.1002/fsn3.70645 (PMC12267880; doi:10.1002/fsn3.70645)
Supplement: Supplementary file 1 — Data S1. [file FSN3-13-e70645-s001.docx]

**Supplementary data**

Table S1. Summary of nutritional values

| Index | Full Name | Calculation Formula |
| --- | --- | --- |
| $\frac{\mathrm{PUFA}}{\mathrm{SFA}}$ | Polyunsaturated fatty acid/ saturated fatty acid ratio | $\frac{\sum PUFA}{\sum SFA}$ |
| $\frac{\omega6}{\sum\omega3}$ | Omega-6/omega-3 ratio | $\frac{\sum\omega6}{\sum\omega3}$ |
| UI | Unsaturation index | 1*x* (monoenoics %) + 2*(dienoics %) + 3* (trienoics%) + 4 *x* (tetraenoics%) + 5*( pentaenoics %) + 6 *x* (hexaenoics %) |
| AI | Atherogenic index | $\frac{[C12:0 + (4* C14:0) + C16:0]}{\sum SUFA}$ |
| TI | Thrombogenic index | $(C14:0+C16:0+C18:0)/([(0.5 x \sum MUFA)+(0.5 x\sum\omega6 PUFA)+(3x \sum\omega3 PUFA)+(\omega3/\omega6)])$ |
| $\frac{H}{H}$ | Hypocholesterolemic/ Hypercholesterolemic ratio | $\frac{(cis-C18:1 + \sum PUFA)}{(C12:0+C14:0+C16:0)}$ |

Table S2. Kruskal-Wallis test for moisture of tested seaweeds (brown -green and red spp.).

| Depend.  Moisture | Multiple Comparisons z´ values: Moisture (Spreadsheet 1):  Independent (grouping) variable: group Kruskal-Wallis test: H (2, N=51) =34.83414 p=.0000 | | | |
| --- | --- | --- | --- | --- |
|  | B  R:28.833 | G  R:41:583 | R  R:14.981 |  |
| B |  | 1.819367 | 2.064487 |  |
| G | 1.819367 |  | 5.880681 |  |
| R | 2.064487 | 5.880681 |  |  |

| Depend.  Moisture | Multiple Comparisons p values (2-tailed): Moisture  Independent (grouping) variable: group Kruskal-Wallis test: H (2, N=51) =34.83414 p=.0000 | | | |
| --- | --- | --- | --- | --- |
|  | B  R:28.833 | G  R:41:583 | R  R:14.981 |  |
| B |  |  | 0.1169 |  |
| G | 0.2065 |  | 0.0000 |  |
| R | 0.1169 | 0.0000 |  |  |

Table S3. Kruskal- Wallis test for ash of tested seaweeds (brown-green and red spp.).

| Depend.  Ash | Multiple Comparisons z´ values: Ash (Spreadsheet 1)  Independent (grouping) variable: group Kruskal-Wallis test: H (2, N=51) =10.38192 p=.0056 | | | |
| --- | --- | --- | --- | --- |
|  | B  R:10.583 | G  R:23.306 | R  R:31.222 |  |
| B |  | 1.815403 | 3.076031 |  |
| G | 1.815403 |  | 1.750081 |  |
| R | 3.07601 | 1.760081 |  |  |

| Depend.  Ash | Multiple Comparisons p values (2-tailed): Ash (Spreadsheet1) Independent (grouping) variable: group  Kruskal-Wallis test: H (2, N=51) =10.38192 p=.0056 | | | |
| --- | --- | --- | --- | --- |
|  | B  R:10.583 | G  R:23.306 | R  R:31.222 |  |
| B |  | 0.208386 | 0.006293 |  |
| G | 0.208386 |  | 0.240313 |  |
| R | 0.00629 | 0.240313 |  |  |

Table S4. Kruskal- Wallis test for lipids of tested seaweeds (brown-green and red spp.).

| Depend.  Lipids | Multiple Comparisons z´ values: Lipids (Spreadsheet 1)  Independent (grouping) variable: group Kruskal-Wallis test: H (2, N=51) =1.210378 p=.5460 | | | |
| --- | --- | --- | --- | --- |
|  | B  R:27.833 | G  R:28.611 | R  R:23.52 |  |
| B |  | 0.110985 | 0,593402 |  |
| G | 0.110985 |  | 1.052095 |  |
| R | 0,593402 | 1.052095 |  |  |

‘

| Depend.  Lipids | Multiple Comparisons p values (2-tailed): Lipids (Spreadsheet1) Independent (grouping) variable: group Kruskal-Wallis test: H (2, N=51) =1.210378 p=.5460 | | | |
| --- | --- | --- | --- | --- |
|  | B  R:27.833 | G  R:28.611 | R  R:23.52 |  |
| B |  | 1.000000 | 1.00000 |  |
| G | 1.000000 |  | 0.878267 |  |
| R | 1.000000 | 0.878267 |  |  |

Table S5. Kruskal- Wallis test for carbohydrates of tested seaweeds (brown -green and red spp.).

| Depend.  CHCO | Multiple Comparisons z´ values: CHCO (Spreadsheet 1)  Independent (grouping) variable: group Kruskal-Wallis test: H (2, N=51) =.6233733 p=.7322 | | | |
| --- | --- | --- | --- | --- |
|  | B  R:30.500 | G  R:25.444 | R  R:25.370 |  |
| B |  | 0.721405 | 0.764523 |  |
| G | 0.721405 |  | 0.016375 |  |
| R | 0.764523 | 0.016375 |  |  |

| Depend.  CHCO | Multiple Comparisons p values (2-tailed): CHCO (Spreadsheet1) Independent (grouping) variable: group  Kruskal-Wallis test: H (2, N=51) =.6233733 p=.7322 | | | |
| --- | --- | --- | --- | --- |
|  | B  R:30.500 | G  R:25.444 | R  R:25.370 |  |
| B |  | 1.000000 | 1.00000 |  |
| G | 1.000000 |  | 1.000000 |  |
| R | 1.000000 | 1.000000 |  |  |

Table S6. Kruskal- Wallis test for proteins of tested seaweeds (brown -green and Red spp.).

| Depend.  Proteins | Multiple Comparisons z´ values: Proteins (Spreadsheet 1)  Independent (grouping) variable: group Kruskal-Wallis test: H (2, N=51) =9.274343 p=.0097 | | | |
| --- | --- | --- | --- | --- |
|  | B  R:11.833 | G  R:32.667 | R  R:24.704 |  |
| B |  | 2.972822 | 1.918207 |  |
| G | 2.972822 |  | 1.760315 |  |
| R | 1.918207 | 1.760315 |  |  |

| Depend.  Proteins | Multiple Comparisons p values (2-tailed): Proteins (Spreadsheet1) Independent (grouping) variable: group  Kruskal-Wallis test: H (2, N=51) =9.274343 p=.0097 | | | |
| --- | --- | --- | --- | --- |
|  | B  R:11.833 | G  R:32.667 | R  R:24.704 |  |
| B |  | 0.008852 | 0.165254 |  |
| G | 0.008852 |  | 0.235063 |  |
| R | 0.165254 | 0.335063 |  |  |

Table S7. Kruskal- Wallis test for calories of tested seaweeds (brown -green and red spp.).

| Depend.  Cal. | Multiple Comparisons z´ values: Calories (Spreadsheet 1)  Independent (grouping) variable: group Kruskal-Wallis test: H (2, N=51) =4.234732 p=.1203 | | | |
| --- | --- | --- | --- | --- |
|  | B  R:20.500 | G  R:31.667 | R  R:23.444 |  |
| B |  | 1.593433 | 0.438842 |  |
| G | 1.593433 |  | 1.817628 |  |
| R | 0.438842 | 1.8176128 |  |  |

| Depend.  Cal. | Multiple Comparisons p values (2-tailed): Calories (Spreadsheet1) Independent (grouping) variable: group  Kruskal-Wallis test: H (2, N=51) =4.234732 p=.1203 | | | |
| --- | --- | --- | --- | --- |
|  | B  R:20.500 | G  R:31.667 | R  R:23.444 |  |
| B |  | 0.333190 | 1.00000 |  |
| G | 0.333190 |  | 0.207363 |  |
| R | 1.000000 | 0.207363 |  |  |

Table S8. Kruskal- Wallis test for Chlorophyll *a* of tested seaweeds (brown- green and red spp.).

| Depend.  Chl *a* | Multiple Comparisons z´ values: Chl *a* (Spreadsheet 1)  Independent (grouping) variable: group Kruskal-Wallis test: H (2, N=51) =8.011172 p=.0182 | | | |
| --- | --- | --- | --- | --- |
|  | B  R:21.083 | G  R:33.972 | R  R:21.778 |  |
| B |  | 1.839186 | 0.103500 |  |
| G | 1.839186 |  | 2.695738 |  |
| R | 0.103500 | 2.695738 |  |  |

| Depend.  Chl *a* | Multiple Comparisons p values (2-tailed): Chl *a*  Independent (grouping) variable: group Kruskal-Wallis test: H (2, N=51) =8.011172 p=.0182 | | | |
| --- | --- | --- | --- | --- |
|  | B  R:21.083 | G  R:33.972 | R  R:21.778 |  |
| B |  |  | 1.00000 |  |
| G | 0.197664 |  | 0.021070 |  |
| R | 1.00000 | 0.021070 |  |  |

Table S9. Kruskal-Wallis test for Total chlorophylls of tested seaweeds (brown-green and red spp.).

| Depend.  T Chl | Multiple Comparisons z´ values: T Chl (Spreadsheet 1)  Independent (grouping) variable: group Kruskal-Wallis test: H (2, N=51) =6.468116 p=.0394 | | | |
| --- | --- | --- | --- | --- |
|  | B  R:19.417 | G  R:33.028 | R  R:22.778 |  |
| B |  | 1.942244 | 0.500942 |  |
| G | 1.942244 |  | 2.265894 |  |
| R | 0.500942 | 2.265894 |  |  |

| Depend.  T. Chl | Multiple Comparisons p values (2-tailed): T. Chl. (Spreadsheet1) Independent (grouping) variable: group  Kruskal-Wallis test: H (2, N=51) =6.468116 p=.0394 | | | |
| --- | --- | --- | --- | --- |
|  | B  R:19.417 | G  R:33.028 | R  R:22.778 |  |
| B |  | 0.156323 | 1.000000 |  |
| G | 0.156323 |  | 0.070374 |  |
| R | 1.000000 | 0.070374 |  |  |

Table S10. Kruskal-Wallis test for carotenoid of tested seaweeds (brown-green and red spp.).

| Depend.  Car. | Multiple Comparisons z´ values: Car. (Spreadsheet 1)  Independent (grouping) variable: group Kruskal-Wallis test: H (2, N=51) =2.597822 p=.2728 | | | |
| --- | --- | --- | --- | --- |
|  | B  R:24.250 | G  R:30.528 | R  R:23.370 |  |
| B |  | 0.895810 | 0.131100 |  |
| G | 0.895810 |  | 1.582237 |  |
| R | 0.131100 | 1.582237 |  |  |

| Depend.  Car. | Multiple Comparisons p values (2-tailed): Car. (Spreadsheet1) Independent (grouping) variable: group Kruskal-Wallis test: H (2, N=51) =2.597822 p=.2728 | | | |
| --- | --- | --- | --- | --- |
|  | B  R:24.250 | G  R:30.528 | R  R:23.370 |  |
| B |  | 1.000000 | 1.000000 |  |
| G | 1.000000 |  | 0.340787 |  |
| R | 1.000000 | 0.340787 |  |  |

Table S11. Kruskal-Wallis test for Vitamin C of tested seaweeds (brown-green and red spp.).

| Depend.  Vit c | Multiple Comparisons z´ values: Vit. C (Spreadsheet 1)  Independent (grouping) variable: group Kruskal-Wallis test: H (2, N=51) =.3417492 p=.8429 | | | |
| --- | --- | --- | --- | --- |
|  | B  R:19.417 | G  R:33.028 | R  R:22.778 |  |
| B |  | 0.582673 | 0.485762 |  |
| G | 0.582673 |  | 0.182172 |  |
| R | 0.485762 | 0.182172 |  |  |

| Depend.  Vit. C | Multiple Comparisons p values (2-tailed): Vit. C (Spreadsheet1) Independent (grouping) variable: group  Kruskal-Wallis test: H (2, N=51) =3417492 p=.8429 | | | |
| --- | --- | --- | --- | --- |
|  | B  R:19.417 | G  R:33.028 | R  R:22.778 |  |
| B |  | 1.000000 | 1.000000 |  |
| G | 1.000000 |  | 1.00000 |  |
| R | 1.000000 | 1.000000 |  |  |

Table S12. Kruskal-Wallis test for Vitamin E of tested seaweeds (brown- green and red spp.).

| Depend.  Vit. E | Multiple Comparisons z´ values: Vit. E (Spreadsheet 1)  Independent (grouping) variable: group Kruskal-Wallis test: H (2, N=51) =1.018045 p=.6011 | | | |
| --- | --- | --- | --- | --- |
|  | B  R:30.500 | G  R:23.694 | R  R:26.537 |  |
| B |  | 0.971122 | 0.590642 |  |
| G | 0.971122 |  | 0.628392 |  |
| R | 0.590642 | 0.628392 |  |  |

| Depend.  Vit E | Multiple Comparisons p values (2-tailed): Vit. E (Spreadsheet1) Independent (grouping) variable: group  Kruskal-Wallis test: H (2, N=51) =1.018045 p=.6011 | | | |
| --- | --- | --- | --- | --- |
|  | B  R:30.500 | G  R:23.694 | R  R:26.537 |  |
| B |  | 0.994463 | 1.000000 |  |
| G | 0.994463 |  | 1000000 |  |
| R | 1.000000 | 1000000 |  |  |

Table S13. Kruskal-Wallis test for Niacin of tested seaweeds (brown- green and red spp.).

| Depend.  Niacin | Multiple Comparisons z´ values: Niacin (Spreadsheet 1)  Independent (grouping) variable: group Kruskal-Wallis test: H (2, N=51) =3.573717 p=.1673 | | | |
| --- | --- | --- | --- | --- |
|  | B  R:36.250 | G  R:26.222 | R  R:23.574 |  |
| B |  | 1.430918 | 1.889227 |  |
| G | 1.430918 |  | 0.585407 |  |
| R | 1.889227 | 0.585407 |  |  |

| Depend.  Niacin | Multiple Comparisons p values (2-tailed): Niacin (Spreadsheet1) Independent (grouping) variable: group  Kruskal-Wallis test: H (2, N=51) =3.573717 p=.1673 | | | |
| --- | --- | --- | --- | --- |
|  | B  R:36.250 | G  R:26.222 | R  R:23.574 |  |
| B |  | 0.457361 | 0.176584 |  |
| G | 0.457361 |  | 1.000000 |  |
| R | 0.176584 | 1.000000 |  |  |

Table S14. Kruskal-Wallis test for β-carotene of tested seaweeds (brown- green and red spp.).

| Depend.  β-carotene | Multiple Comparisons z´ values: β-carotene (Spreadsheet 1) Independent (grouping) variable: group Kruskal-Wallis test: H (2, N=51) =.3328908 p=.8467 | | | |
| --- | --- | --- | --- | --- |
|  | B  R:27.833 | G  R:27.056 | R  R:24.889 |  |
| B |  | 0.110985 | 0.438842 |  |
| G | 0.110985 |  | 0.478969 |  |
| R | 0.438842 | 0.478969 |  |  |

| Depend.  β-carotene | Multiple Comparisons p values (2-tailed): β-carotene (Spreadsheet1) Independent (grouping) variable: group Kruskal-Wallis test: H (2, N=51) ==.3328908 p=.8467 | | | |
| --- | --- | --- | --- | --- |
|  | B  R:27.833 | G  R:27.056 | R  R:24.889 |  |
| B |  | 1.000000 | 1.000000 |  |
| G | 1.000000 |  | 1.000000 |  |
| R | 1.000000 | 1.000000 |  |  |

Table S15. Kruskal-Wallis test for lycopene of tested seaweeds (brown- green and red spp.).

| Depend.  Lycopene | Multiple Comparisons z´ values: Lycopene (Spreadsheet 1)  Independent (grouping) variable: group Kruskal-Wallis test: H (2, N=51) =6.468116 p=.0394 | | | |
| --- | --- | --- | --- | --- |
|  | B  R:31.833 | G  R:26.111 | R  R:24.630 |  |
| B |  | 0.816535 | 1.073644 |  |
| G | 0.816535 |  | 0.327501 |  |
| R | 1.073644 | 0.327501 |  |  |

| Depend.  Lycopene | Multiple Comparisons p values (2-tailed): Lycopene (Spreadsheet1) Independent (grouping) variable: group Kruskal-Wallis test: H (2, N=51) =6.468116 p=.0394 | | | |
| --- | --- | --- | --- | --- |
|  | B  R:31.833 | G  R:26.111 | R  R:24.630 |  |
| B |  | 1.000000 | 0.848947 |  |
| G | 1.000000 |  | 1.000000 |  |
| R | 0.848947 | 1.000000 |  |  |

Table S16. Kruskal-Wallis test for moisture of tested seaweeds along Alexandria coast.

| Depend.:  Moisture | Multiple Comparisons z´ values: Moisture (Spreadsheet 1) Independent (grouping) variable: site Kruskal-Wallis test: H (6, N=51) =8.906194 p=.1789 | | | | | | |
| --- | --- | --- | --- | --- | --- | --- | --- |
|  | Abo Qir  R:24.531 | Gleem  R:19.278 | Sidi bashr  R:17.083 | Stanly  R:30.000 | E.H  R:23.876. | El Mex:  R=11.877 | DK  R=12.9765 |
| Abo Qir |  | 0.848128 | 1.0465557 | 0.658062 | 0.979226 | 1.490362 | 1.185586 |
| Gleem | 0.848128 |  | 0.280078 | 1.200240 | 1.559931 | 2.055374 | 1.589509 |
| Sidi bashr | 1.0465557 | 0.280078 |  | 1.346046 | 1.679691 | 2.122621 | 1.702629 |
| Stanly | 0.658062 | 1.200240 | 1.346046 |  | 0.156315 | 0.453119 | 0.60197 |
| E.H. | 0.979226 | 1.559931 | 1.679691 | 0.156315 |  | 0.876536 | 0.54325 |
| El Mex | 1.490362 | 2.055374 | 2.122621 | 0.453119 | 0.545365 |  | 0.76546 |
| DK | 1.185586 | 1.589509 | 1.702629 | 0.60197 | 0.342157 | 0.543457 |  |

| Depend.:  Moisture | Multiple Comparisons p values (2-tailed): Moisture (Spreadsheet1) Independent (grouping) variable: group Kruskal-Wallis test: H (6, N=51) =8.906194 p=.1789 | | | | | | |
| --- | --- | --- | --- | --- | --- | --- | --- |
|  | Abo Qir  R:24.531 | Gleem  R:19.278 | Sidi bashr  R:17.083 | Stanly  R:30.000 | E.H  R:23.876. | El Mex:  R=11.877 | DK  R=12.9765 |
| Abo Qir |  | 1.000000 | 1.000000 | 1.000000 | 1.000000 | 1.000000 | 1.000000 |
| Gleem | 1.000000 |  | 1.000000 | 1.000000 | 1.000000 | 0.836701 | 1.000000 |
| Sidi bashr | 1.000000 | 1.000000 |  | 1.000000 | 1.000000 | 0.709497 | 1.000000 |
| Stanly | 1.000000 | 1.000000 | 1.000000 |  | 1.000000 | 1.000000 | 1.000000 |
| E.H. | 1.000000 | 1.000000 | 1.000000 | 1.000000 |  | 1.000000 | 1.000000 |
| El Mex | 1.000000 | 0.836701 | 0.709497 | 1.000000 | 1.000000 |  | 0.76546 |
| DK | 1.000000 | 1.000000 | 1.000000 | 1.000000 | 1.000000 | 1.000000 |  |

Table S17. Kruskal-Wallis test for ash of tested seaweeds along Alexandria coast.

| Depend.:  Ash | Multiple Comparisons z´ values: Ash (Spreadsheet 1) Independent (grouping) variable: site Kruskal-Wallis test: H (6, N=51) =5.702897 p=.4573 | | | | | | |
| --- | --- | --- | --- | --- | --- | --- | --- |
|  | Abo Qir  R:20.406 | Gleem  R:24.667 | Sidi bashr  R:31.917 | Stanly  R:32.750 | E.H  R:20.765 | El Mex:  R=15.987 | DK  R=15.765 |
| Abo Qir |  | 0.687808 | 1.61407 | 1.485340 | 0.949952 | 0.985483 | 1.667668 |
| Gleem | 0.687808 |  | 0.925322 | 0.904844 | 1.559931 | 2.055374 | 1.589509 |
| Sidi bashr | 1.61407 | 0.925322 |  | 0.086842 | 0.553442 | 0.643533 | 0.583562 |
| Stanly | 1.485340 | 0.904844 | 0.086842 |  | 0.156315 | 0.453119 | 0.485460 |
| E.H. | 0.949952 | 1.559931 | 0.553442 | 0.581839 |  | 0.876536 | 1.04325 |
| El Mex | 0.985483 | 2.055374 | 0.643533 | 0.659082 | 1.87532 |  | 0.77656 |
| DK | 1.667668 | 1.589509 | 0.583562 | 0.485460 | 2.98765 | 0.585437 |  |

| Depend.:  Ash | Multiple Comparisons p values (2-tailed): Ash (Spreadsheet1) Independent (grouping) variable: group Kruskal-Wallis test: H (6, N=51) =5.702897 p=.4573 | | | | | | |
| --- | --- | --- | --- | --- | --- | --- | --- |
|  | Abo Qir  R:20.406 | Gleem  R:24.667 | Sidi bashr  R:31.917 | Stanly  R:32.750 | E.H  R:20.765 | El Mex:  R=15.987 | DK  R=15.765 |
| Abo Qir |  | 1.000000 | 1.000000 | 1.000000 | 1.000000 | 1.000000 | 1.000000 |
| Gleem | 1.000000 |  | 1.000000 | 1.000000 | 1.000000 | 1.000000 | 1.000000 |
| Sidi bashr | 1.000000 | 1.000000 |  | 1.000000 | 1.000000 | 1.000000 | 1.000000 |
| Stanly | 1.000000 | 1.000000 | 1.000000 |  | 1.000000 | 1.000000 | 1.000000 |
| E.H. | 1.000000 | 1.000000 | 1.000000 | 1.000000 |  | 1.000000 | 1.000000 |
| El Mex | 1.000000 | 1.000000 | 1.000000 | 1.000000 | 1.000000 |  | 1.000000 |
| DK | 1.000000 | 1.000000 | 1.000000 | 1.000000 | 1.000000 | 1.000000 |  |

Table S18. Kruskal-Wallis test for lipids of tested seaweeds along Alexandria coast.

| Depend.:  Lipids | Multiple Comparisons z´ values: Lipids (Spreadsheet 1) Independent (grouping) variable: site Kruskal-Wallis test: H (6, N=51) =8.973634 p=.1751 | | | | | | |
| --- | --- | --- | --- | --- | --- | --- | --- |
|  | Abo Qir  R:22.125 | Gleem  R:19.000 | Sidi bashr  R:35.667 | Stanly  R:30.500 | E.H  R:19.278 | El Mex:  R=11.445 | DK  R=24.531 |
| Abo Qir |  | 0.504505 | 1.902831 | 1.007775 | 0.146372 | 1.301032 | 1.692893 |
| Gleem | 0.504505 |  | 2.127178 | 1.287304 | 0.531795 | 1.592002 | 1.893069 |
| Sidi bashr | 1.902831 | 2.127178 |  | 0.538418 | 1.456379 | 0.643533 | 0.439388 |
| Stanly | 1.007775 | 1.287304 | 0.538418 |  | 0.764207 | 0.000000 | 0.815572 |
| E.H. | 0.146372 | 0.531795 | 1.456379 | 0.764207 |  | 3.654335 | 1.543258 |
| El Mex | 1.301032 | 1.592002 | 0.643533 | 0.000000 | 3.654335 |  | 1.76445 |
| DK | 1.692893 | 1.893069 | 0.439388 | 0.815572 | 1.543258 | 1.76445 |  |

| Depend.:  Lipids | Multiple Comparisons p values (2-tailed): Lipids (Spreadsheet1) Independent (grouping) variable: group Kruskal-Wallis test: H (6, N=51) =8.973634 p=.1751 | | | | | | |
| --- | --- | --- | --- | --- | --- | --- | --- |
|  | Abo Qir  R:22.125 | Gleem  R:19.000 | Sidi bashr  R:35.667 | Stanly  R:30.500 | E.H  R: 19.278 | El Mex:  R=11.445 | DK  R=24.531 |
| Abo Qir |  | 1.000000 | 1.000000 | 1.000000 | 1.000000 | 1.000000 | 1.000000 |
| Gleem | 1.000000 |  | 0.701511 | 1.000000 | 1.000000 | 1.000000 | 1.000000 |
| Sidi bashr | 1.000000 | 0.701511 |  | 1.000000 | 1.000000 | 1.000000 | 1.000000 |
| Stanly | 1.000000 | 1.000000 | 1.000000 |  | 1.000000 | 1.000000 | 1.000000 |
| E.H. | 1.000000 | 1.000000 | 1.000000 | 1.000000 |  | 1.000000 | 1.000000 |
| El Mex | 1.000000 | 1.000000 | 1.000000 | 1.000000 | 1.000000 |  | 1.000000 |
| DK | 1.000000 | 1.000000 | 1.000000 | 1.000000 | 1.000000 | 1.000000 |  |

Table S19. Kruskal-Wallis test for carbohydrates of tested seaweeds along Alexandria coast.

| Depend.:  CHCO | Multiple Comparisons z´ values: Carbohydrates (Spreadsheet 1) Independent (grouping) variable: site Kruskal-Wallis test: H (6, N=51) =2.050275 p=.9150 | | | | | | |
| --- | --- | --- | --- | --- | --- | --- | --- |
|  | Abo Qir  R:29.313 | Gleem  R:24.111 | Sidi bashr  R:21.333 | Stanly  R:27.250 | E.H  R:13.765 | El Mex:  R=22.877 | DK  R=15.8765 |
| Abo Qir |  | 0.839720 | 1.121207 | 0.248183 | 1.027529 | 0.359240 | 0.162563 |
| Gleem | 0.839720 |  | 0.354530 | 0.351366 | 0.269443 | 0.399923 | 0.291609 |
| Sidi bashr | 1.121207 | 0.354530 |  | 0.616576 | 0.077674 | 0.705811 | 0.508042 |
| Stanly | 0.248183 | 0.351366 | 0.616576 |  | 0.547103 | 0.027462 | 0.019418 |
| E.H. | 1.027529 | 0.269443 | 0.077674 | 0.547103 |  | 0.875875 | 0.654382 |
| El Mex | 0.359240 | 0.399923 | 0.705811 | 0.027462 | 0.875875 |  | 0.876565 |
| DK | 0.162563 | 0.291609 | 0.508042 | 0.019418 | 0.654382 | 0.876565 |  |

| Depend.:  CHCO | Multiple Comparisons p values (2-tailed): CHCO (Spreadsheet1) Independent (grouping) variable: group Kruskal-Wallis test: H (6, N=51) =2.050275 p=.9150 | | | | | | |
| --- | --- | --- | --- | --- | --- | --- | --- |
|  | Abo Qir  R:29.313 | Gleem  R:24.111 | Sidi bashr  R:21.333 | Stanly  R:27.250 | E.H  R:13.765 | El Mex:  R=22.877 | DK  R=15.8765 |
| Abo Qir |  | 1.000000 | 1.000000 | 1.000000 | 1.000000 | 1.000000 | 1.000000 |
| Gleem | 1.000000 |  | 1.000000 | 1.000000 | 1.000000 | 1.000000 | 1.000000 |
| Sidi bashr | 1.000000 | 1.000000 |  | 1.000000 | 1.000000 | 1.000000 | 1.000000 |
| Stanly | 1.000000 | 1.000000 | 1.000000 |  | 1.000000 | 1.000000 | 1.000000 |
| E.H. | 1.000000 | 1.000000 | 1.000000 | 1.000000 |  | 1.000000 | 1.000000 |
| El Mex | 1.000000 | 1.000000 | 1.000000 | 1.000000 | 1.000000 |  | 1.000000 |
| DK | 1.000000 | 1.000000 | 1.000000 | 1.000000 | 1.000000 | 1.000000 |  |

Table S20. Kruskal-Wallis test for proteins of tested seaweeds along Alexandria coast.

| Depend.:  Proteins | Multiple Comparisons z´ values: Proteins (Spreadsheet 1) Independent (grouping) variable: site Kruskal-Wallis test: H (6, N=51) =8.973382 p=.1751 | | | | | | |
| --- | --- | --- | --- | --- | --- | --- | --- |
|  | Abo Qir  R:21.500 | Gleem  R:21.889 | Sidi bashr  R:35.000 | Stanly  R:21.750 | E.H  R:20.876. | El Mex:  R=18.765 | DK  R=22.432 |
| Abo Qir |  | 0.062783 | 1.896976 | 0.030083 | 0.866520 | 1.087430 | 2.287087 |
| Gleem | 0.062783 |  | 1.673380 | 0.015547 | 0.737422 | 0.915209 | 2.160776 |
| Sidi bashr | 1.896976 | 1.673380 |  | 1.380783 | 0.854409 | 0.809606 | 0.988623 |
| Stanly | 0.030083 | 0.015547 | 1.380783 |  | 0.616576 | 0.741467 | 1.961257 |
| E.H. | 0.866520 | 0.737422 | 0.854409 | 0.616576 |  | 0.876549 | 0.873454 |
| El Mex | 1.087430 | 0.915209 | 0.809606 | 0.741467 | 0.876549 |  | 1.987545 |
| DK | 2.287087 | 2.160776 | 0.988623 | 1.961257 | 0.873454 | 1.987545 |  |

| Depend.:  Proteins | Multiple Comparisons p values (2-tailed): Proteins (Spreadsheet1) Independent (grouping) variable: group Kruskal-Wallis test: H (6, N=51) =8.973383 p=.1751 | | | | | | |
| --- | --- | --- | --- | --- | --- | --- | --- |
|  | Abo Qir  R:21.500 | Gleem  R:21.889 | Sidi bashr  R:35.000 | Stanly  R:21.750 | E.H  R:20.876. | El Mex:  R=18.765 | DK  R=22.432 |
| Abo Qir |  | 1.000000 | 1.000000 | 1.000000 | 1.000000 | 1.000000 | 0.466005 |
| Gleem | 1.000000 |  | 1.000000 | 1.000000 | 1.000000 | 1.000000 | 0.644966 |
| Sidi bashr | 1.000000 | 1.000000 |  | 1.000000 | 1.000000 | 1.000000 | 1.000000 |
| Stanly | 1.000000 | 1.000000 | 1.000000 |  | 1.000000 | 1.000000 | 1.000000 |
| E.H. | 1.000000 | 1.000000 | 1.000000 | 1.000000 |  | 1.000000 | 1.000000 |
| El Mex | 1.000000 | 1.000000 | 1.000000 | 1.000000 | 1.000000 |  | 1.000000 |
| DK | 0.466005 | 0.644966 | 1.000000 | 1.000000 | 1.000000 | 1.000000 |  |

Table S21. Kruskal-Wallis test for calories of tested seaweeds along Alexandria coast.

| Depend.:  Cal. | Multiple Comparisons z´ values: Calories (Spreadsheet 1) Independent (grouping) variable: site Kruskal-Wallis test: H (6, N=51) =9.253561 p=.1598 | | | | | | |
| --- | --- | --- | --- | --- | --- | --- | --- |
|  | Abo Qir  R:22.875 | Gleem  R:17.556 | Sidi bashr  R:33.500 | Stanly  R:30.250 | E.H  R:16.87 | El Mex:  R=21.876 | DK  R=14.876 |
| Abo Qir |  | 0.858779 | 1.492991 | 1.887444 | 0.275179 | 1.145685 | 1.939540 |
| Gleem | 0.858779 |  | 2.035000 | 1.421010 | 0.928868 | 1.757354 | 2.318532 |
| Sidi bashr | 1.492991 | 2.035000 |  | 0.338683 | 1.009756 | 0.404803 | 0.906238 |
| Stanly | 1.887444 | 1.421010 | 0.338683 |  | 0.564471 | 0.000000 | 1.106848 |
| E.H. | 0.275179 | 0.928868 | 1.009756 | 0.564471 |  | 0.654270 | 2.987544 |
| El Mex | 1.145685 | 1.757354 | 0.404803 | 0.000000 | 0.654270 |  | 2.876435 |
| DK | 1.939540 | 2.318532 | 0.906238 | 1.106848 | 2.987544 | 2.876435 |  |

| Depend.:  Cal. | Multiple Comparisons p values (2-tailed): Calories (Spreadsheet1) Independent (grouping) variable: group Kruskal-Wallis test: H (6, N=51) =9.253561 p=.1598 | | | | | | |
| --- | --- | --- | --- | --- | --- | --- | --- |
|  | Abo Qir  R:22.875 | Gleem  R:17.556 | Sidi bashr  R:33.500 | Stanly  R:30.250 | E.H  R:16.87 | El Mex:  R=21.876 | DK  R=14.876 |
| Abo Qir |  | 1.000000 | 1.000000 | 1.000000 | 1.000000 | 1.000000 | 1.000000 |
| Gleem | 1.000000 |  | 0.878867 | 1.000000 | 1.000000 | 1.000000 | 0.428829 |
| Sidi bashr | 1.000000 | 0.878867 |  | 1.000000 | 1.000000 | 1.000000 | 1.000000 |
| Stanly | 1.000000 | 1.000000 | 1.000000 |  | 1.000000 | 1.000000 | 1.000000 |
| E.H. | 1.000000 | 1.000000 | 1.000000 | 1.000000 |  | 1.000000 | 1.000000 |
| El Mex | 1.000000 | 1.000000 | 1.000000 | 1.000000 | 1.000000 |  | 1.000000 |
| DK | 1.000000 | 0.428829 | 1.000000 | 1.000000 | 1.000000 | 1.000000 |  |

Table S22. Kruskal-Wallis test for chlorophyll *a* of tested seaweeds along Alexandria coast.

| Depend.:  Chl *a* | Multiple Comparisons z´ values: Chl *a* (Spreadsheet 1) Independent (grouping) variable: site Kruskal-Wallis test: H (6, N=51) =1.307971 p=.9712 | | | | | | |
| --- | --- | --- | --- | --- | --- | --- | --- |
|  | Abo Qir  R:27.563 | Gleem  R:24.222 | Sidi bashr  R:22.500 | Stanly  R:25.750 | E.H  R:21.765 | El Mex:  R=21.987 | DK  R=20.765 |
| Abo Qir |  | 0.539259 | 0.711366 | 0.218101 | 0.389349 | 0.339822 | 0.498899 |
| Gleem | 0.539259 |  | 0.219808 | 0.171019 | 0.779965 | 1.159585 | 0.191219 |
| Sidi bashr | 0.711366 | 0.219808 |  | 0.338683 | 0.912664 | 0.358095 | 0.041193 |
| Stanly | 0.218101 | 0.171019 | 0.338683 |  | 0.477629 | 0.041193 | 0.291276 |
| E.H. | 0.389349 | 0.779965 | 0.912664 | 0.477629 |  | 0.876438 | 0.786448 |
| El Mex | 0.339822 | 1.159585 | 0.358095 | 0.041193 | 0.876438 |  | 1.876540 |
| DK | 0.498899 | 0.191219 | 0.041193 | 0.291276 | 0.786448 | 1.876540 |  |

| Depend.:  Chl *a* | Multiple Comparisons p values (2-tailed): Chl *a* (Spreadsheet1) Independent (grouping) variable: group Kruskal-Wallis test: H (6, N=51) =1.307971 p=.9712 | | | | | | |
| --- | --- | --- | --- | --- | --- | --- | --- |
|  | Abo Qir  R:27.563 | Gleem  R:24.222 | Sidi bashr  R:22.500 | Stanly  R:25.750 | E.H  R:21.765 | El Mex:  R=21.987 | DK  R=20.765 |
| Abo Qir |  | 1.000000 | 1.000000 | 1.000000 | 1.000000 | 1.000000 | 1.000000 |
| Gleem | 1.000000 |  | 1.000000 | 1.000000 | 1.000000 | 1.000000 | 1.000000 |
| Sidi bashr | 1.000000 | 1.000000 |  | 1.000000 | 1.000000 | 1.000000 | 1.000000 |
| Stanly | 1.000000 | 1.000000 | 1.000000 |  | 1.000000 | 1.000000 | 1.000000 |
| E.H. | 1.000000 | 1.000000 | 1.000000 | 1.000000 |  | 1.000000 | 1.000000 |
| El Mex | 1.000000 | 1.000000 | 1.000000 | 1.000000 | 1.000000 |  | 1.000000 |
| DK | 1.000000 | 1.000000 | 1.000000 | 1.000000 | 1.000000 | 1.000000 |  |

Table S23. Kruskal-Wallis test for total Chlorophylls of tested seaweeds along Alexandria coast.

| Depend.:  T Chl | Multiple Comparisons z´ values: Total chlorophylls (Spreadsheet 1) Independent (grouping) variable: site Kruskal-Wallis test: H (6, N=51) =5.373358 p=.4969 | | | | | | |
| --- | --- | --- | --- | --- | --- | --- | --- |
|  | Abo Qir  R:22.375 | Gleem  R:27.667 | Sidi bashr  R:26.167 | Stanly  R:28.250 | E.H  R:20.876 | El Mex:  R=21.987 | DK  R=20.876 |
| Abo Qir |  | 0.854294 | 0.532793 | 0.706947 | 1.961380 | 0.466041 | 0.751151 |
| Gleem | 0.854294 |  | 0.191446 | 0.065298 | 1.106133 | 0.317247 | 1.175997 |
| Sidi bashr | 0.532793 | 0.191446 |  | 0.217104 | 1.184522 | 0.098606 | 1.002354 |
| Stanly | 0.706947 | 0.065298 | 0.217104 |  | 0.842364 | 0.315810 | 1.106848 |
| E.H. | 1.961380 | 1.106133 | 1.184522 | 0.842364 |  | 0.436780 | 0.765308 |
| El Mex | 0.466041 | 0.317247 | 0.098606 | 0.315810 | 0.436780 |  | 2.876439 |
| DK | 0.751151 | 1.175997 | 1.002354 | 1.106848 | 0.765308 | 2.876439 |  |

| epend.:  T. Chl | Multiple Comparisons p values (2-tailed): Total chlorophylls (Spreadsheet1) Independent (grouping) variable: group Kruskal-Wallis test: H (6, N=51) =55.373358 p=.4969 | | | | | | |
| --- | --- | --- | --- | --- | --- | --- | --- |
|  | Abo Qir  R:22.375 | Gleem  R:27.667 | Sidi bashr  R:26.167 | Stanly  R:28.250 | E.H  R:20.876 | El Mex:  R=21.987 | DK  R=20.876 |
| Abo Qir |  | 1.000000 | 1.000000 | 1.000000 | 1.000000 | 1.000000 | 1.000000 |
| Gleem | 1.000000 |  | 1.000000 | 1.000000 | 1.000000 | 1.000000 | 1.000000 |
| Sidi bashr | 1.000000 | 1.000000 |  | 1.000000 | 1.000000 | 1.000000 | 1.000000 |
| Stanly | 1.000000 | 1.000000 | 1.000000 |  | 1.000000 | 1.000000 | 1.000000 |
| E.H. | 1.000000 | 1.000000 | 1.000000 | 1.000000 |  | 1.000000 | 1.000000 |
| El Mex | 1.000000 | 1.000000 | 1.000000 | 1.000000 | 1.000000 |  | 1.000000 |
| DK | 1.000000 | 1.000000 | 1.000000 | 1.000000 | 1.000000 | 1.000000 |  |

Table S24. Kruskal-Wallis test for carotenoids of tested seaweeds along Alexandria coast.

| Depend.:  Car. | Multiple Comparisons z´ values: Carotenoids (Spreadsheet 1) Independent (grouping) variable: site Kruskal-Wallis test: H (6, N=51) =4.820574 p=.5670 | | | | | | |
| --- | --- | --- | --- | --- | --- | --- | --- |
|  | Abo Qir  R:29.438 | Gleem  R:25.778 | Sidi bashr  R:26.833 | Stanly  R:24.500 | E.H  R:21.655 | El Mex:  R=21.765 | DK  R=212.931 |
| Abo Qir |  | 0.590831 | 0.365929 | 0.594136 | 0.201993 | 1.019466 | 2.057258 |
| Gleem | 0.590831 |  | 0.134721 | 0.143034 | 0.283624 | 0.401846 | 1.658826 |
| Sidi bashr | 0.365929 | 0.134721 |  | 0.243157 | 0.135929 | 0.493029 | 1.675167 |
| Stanly | 0.594136 | 0.143034 | 0.243157 |  | 0.364735 | 0.178501 | 1.398124 |
| E.H. | 0.201993 | 0.283624 | 0.135929 | 0.364735 |  | 0.876498 | 0.876439 |
| El Mex | 1.019466 | 0.401846 | 0.493029 | 0.178501 | 0.876498 |  | 2.876476 |
| DK | 2.057258 | 1.658826 | 1.675167 | 1.398124 | 0.876439 | 2.876476 |  |

| Depend.:  Car. | Multiple Comparisons p values (2-tailed): Carotenoids (Spreadsheet1) Independent (grouping) variable: group Kruskal-Wallis test: H (6, N=51) =4.820574 p=.5670 | | | | | | |
| --- | --- | --- | --- | --- | --- | --- | --- |
|  | Abo Qir  R:29.438 | Gleem  R:25.778 | Sidi bashr  R:26.833 | Stanly  R:24.500 | E.H  R:21.655 | El Mex:  R=21.765 | DK  R=212.931 |
| Abo Qir |  | 1.000000 | 1.000000 | 1.000000 | 1.000000 | 1.000000 | 0.832890 |
| Gleem | 1.000000 |  | 1.000000 | 1.000000 | 1.000000 | 1.000000 | 1.000000 |
| Sidi bashr | 1.000000 | 1.000000 |  | 1.000000 | 1.000000 | 1.000000 | 1.000000 |
| Stanly | 1.000000 | 1.000000 | 1.000000 |  | 1.000000 | 1.000000 | 1.000000 |
| E.H. | 1.000000 | 1.000000 | 1.000000 | 1.000000 |  | 1.000000 | 1.000000 |
| El Mex | 1.000000 | 1.000000 | 1.000000 | 1.000000 | 1.000000 |  | 1.000000 |
| DK | 0.832890 | 1.000000 | 1.000000 | 1.000000 | 1.000000 | 1.000000 |  |

Table S25. Kruskal-Wallis test for vitamin C of tested seaweeds along Alexandria coast.

| Depend.:  Vit. C | Multiple Comparisons z´ values: Vitamin C (Spreadsheet 1) Independent (grouping) variable: site Kruskal-Wallis test: H (6, N=51) =8.612224 p=.1966 | | | | | | |
| --- | --- | --- | --- | --- | --- | --- | --- |
|  | Abo Qir  R:30.500 | Gleem  R:21.889 | Sidi bashr  R:19.917 | Stanly  R:32.125 | E.H  R:22.768 | El Mex:  R=21.765 | DK  R=22.766 |
| Abo Qir |  | 1.390190 | 1.487136 | 0.195538 | 0.771842 | 1.747655 | 1.255656 |
| Gleem | 1.390190 |  | 0.251716 | 1.145825 | 0.397073 | 0.36534 | 1.945654 |
| Sidi bashr | 1.487136 | 0.251716 |  | 1.272231 | 0.592261 | 0.083037 | 2.025304 |
| Stanly | 0.195538 | 1.145825 | 1.272231 |  | 0.742496 | 1.414280 | 0.961210 |
| E.H. | 0.771842 | 0.397073 | 0.592261 | 0.742496 |  | 0.987544 | 0.986359 |
| El Mex | 1.747655 | 0.36534 | 0.083037 | 1.414280 | 0.987544 |  | 2.876487 |
| DK | 1.255656 | 1.945654 | 2.025304 | 0.961210 | 0.986359 | 2.876487 |  |

| Depend.:  Vit. C | Multiple Comparisons p values (2-tailed): Vitamin C (Spreadsheet1) Independent (grouping) variable: group Kruskal-Wallis test: H (6, N=51) =8.612224 p=.1966 | | | | | | |
| --- | --- | --- | --- | --- | --- | --- | --- |
|  | Abo Qir  R:30.500 | Gleem  R:21.889 | Sidi bashr  R:19.917 | Stanly  R:32.125 | E.H  R:22.768 | El Mex:  R=21.765 | DK  R=22.766 |
| Abo Qir |  | 1.000000 | 1.000000 | 1.000000 | 1.000000 | 1.000000 | 1.000000 |
| Gleem | 1.000000 |  | 1.000000 | 1.000000 | 1.000000 | 1.000000 | 1.000000 |
| Sidi bashr | 1.000000 | 1.000000 |  | 1.000000 | 1.000000 | 1.000000 | 0.899559 |
| Stanly | 1.000000 | 1.000000 | 1.000000 |  | 1.000000 | 1.000000 | 1.000000 |
| E.H. | 1.000000 | 1.000000 | 1.000000 | 1.000000 |  | 1.000000 | 1.000000 |
| El Mex | 1.000000 | 1.000000 | 1.000000 | 1.000000 | 1.000000 |  | 1.000000 |
| DK | 1.000000 | 1.000000 | 0.899559 | 1.000000 | 1.000000 | 1.000000 |  |

Table S26. Kruskal-Wallis test for vitamin E of tested seaweeds along Alexandria coast.

| Depend.:  Vit. E | Multiple Comparisons z´ values: Vitamin E (Spreadsheet 1) Independent (grouping) variable: site Kruskal-Wallis test: H (6, N=51) =11.72807 p=.0683 | | | | | | |
| --- | --- | --- | --- | --- | --- | --- | --- |
|  | Abo Qir  R:30.469 | Gleem  R:23.778 | Sidi bashr  R:23.083 | Stanly  R:35.000 | E.H  R:33.234 | El Mex:  R=21.543 | DK  R=22.654 |
| Abo Qir |  | 1.080200 | 1.037775 | 0.54552 | 1.588132 | 2.092332 | 1.527528 |
| Gleem | 1.080200 |  | 0.088632 | 1.256210 | 0.588519 | 0.938281 | 2.041264 |
| Sidi bashr | 1.037775 | 0.088632 |  | 1.241836 | 0.456332 | 0.757708 | 2.011573 |
| Stanly | 0.545252 | 1.256210 | 1.241836 |  | 1.649992 | 1.977246 | 0.970920 |
| E.H. | 1.588132 | 0.588519 | 0.456332 | 1.649992 |  | 2.873421 | 1.987643 |
| El Mex | 2.092332 | 0.938281 | 0.757708 | 1.977246 | 2.873421 |  | 3.987476 |
| DK | 1.527528 | 2.041264 | 2.011573 | 0.970920 | 1.987643 | 1.987643 |  |

| Depend.:  Vit. E | Multiple Comparisons p values (2-tailed): Vitamin E (Spreadsheet1) Independent (grouping) variable: group Kruskal-Wallis test: H (6, N=51) =11.72807 p=.0683 | | | | | | |
| --- | --- | --- | --- | --- | --- | --- | --- |
|  | Abo Qir  R:30.469 | Gleem  R:23.778 | Sidi bashr  R:23.083 | Stanly  R:35.000 | E.H  R:33.234 | El Mex:  R=21.543 | DK  R=22.654 |
| Abo Qir |  | 1.000000 | 1.000000 | 1.000000 | 1.000000 | 0.764586 | 1.000000 |
| Gleem | 1.000000 |  | 1.000000 | 1.000000 | 1.000000 | 1.000000 | 0.865717 |
| Sidi bashr | 1.000000 | 1.000000 |  | 1.000000 | 1.000000 | 1.000000 | 0.929564 |
| Stanly | 1.000000 | 1.000000 | 1.000000 |  | 1.000000 | 1.000000 | 1.000000 |
| E.H. | 1.000000 | 1.000000 | 1.000000 | 1.000000 |  | 1.000000 | 1.000000 |
| El Mex | 0.764586 | 1.000000 | 1.000000 | 1.000000 | 1.000000 |  | 1.000000 |
| DK | 1.000000 | 0.865717 | 0.929564 | 1.000000 | 1.000000 | 1.000000 |  |

Table S27. Kruskal-Wallis test for Niacin of tested seaweeds along Alexandria coast.

| Depend.:  Niacin | Multiple Comparisons z´ values: Niacin (Spreadsheet 1) Independent (grouping) variable: site Kruskal-Wallis test: H (6, N=51) =14.04005 p=.0292 | | | | | | |
| --- | --- | --- | --- | --- | --- | --- | --- |
|  | Abo Qir  R:36.125 | Gleem  R:18.111 | Sidi bashr  R:24.333 | Stanly  R:14.250 | E.H  R:15.765 | El Mex:  R=23.876 | DK  R=22.654 |
| Abo Qir |  | 2.908189 | 1.656927 | 2.632249 | 2.218994 | 1.864166 | 0.235435 |
| Gleem | 2.908189 |  | 0.794147 | 0.432211 | 0.283624 | 0.832532 | 1.324192 |
| Sidi bashr | 1.656927 | 0.794147 |  | 1.050784 | 0.466041 | 0.025949 | 0.755198 |
| Stanly | 2.632249 | 0.432211 | 1.050784 |  | 0.633944 | 1.084739 | 1.495216 |
| E.H. | 2.218994 | 0.283624 | 0.466041 | 0.633944 |  | 1.987650 | 1.876535 |
| El Mex | 1.864166 | 0.832532 | 0.025949 | 1.084739 | 1.987650 |  | 3.876423 |
| DK | 0.235435 | 1.324192 | 0.755198 | 1.495216 | 1.876535 | 3.876423 |  |

| Depend.:  Niacin | Multiple Comparisons p values (2-tailed): Niacin (Spreadsheet1) Independent (grouping) variable: group Kruskal-Wallis test: H (6, N=51) =14.04005 p=.0292 | | | | | | |
| --- | --- | --- | --- | --- | --- | --- | --- |
|  | Abo Qir  R:36.125 | Gleem  R:18.111 | Sidi bashr  R:24.333 | Stanly  R:14.250 | E.H  R:15.765 | El Mex:  R=23.876 | DK  R=22.654 |
| Abo Qir |  | 0.076341 | 1.000000 | 0.178126 | 0.556230 | 1.000000 | 1.000000 |
| Gleem | 0.076341 |  | 1.000000 | 1.000000 | 1.000000 | 1.000000 | 1.000000 |
| Sidi bashr | 1.000000 | 1.000000 |  | 1.000000 | 1.000000 | 1.000000 | 1.000000 |
| Stanly | 0.178126 | 1.000000 | 1.000000 |  | 1.000000 | 1.000000 | 1.000000 |
| E.H. | 0.556230 | 1.000000 | 1.000000 | 1.000000 |  | 1.000000 | 1.000000 |
| El Mex | 1.000000 | 1.000000 | 1.000000 | 1.000000 | 1.000000 |  | 1.000000 |
| DK | 1.000000 | 1.000000 | 1.000000 | 1.000000 | 1.000000 | 1.000000 |  |

Table S28. Kruskal-Wallis test for β-carotene of tested seaweeds along Alexandria coast.

| Depend.:  β-carotene | Multiple Comparisons z´ values: β-carotene (Spreadsheet 1) Independent (grouping) variable: site Kruskal-Wallis test: H (6, N=51) =9.249200 p=.1600 | | | | | | |
| --- | --- | --- | --- | --- | --- | --- | --- |
|  | Abo Qir  R:28.469 | Gleem  R:24.500 | Sidi bashr  R:22.750 | Stanly  R:28.625 | E.H  R:19.614 | El Mex:  R=22.245 | DK  R=25.611 |
| Abo Qir |  | 0.640721 | 0.803580 | 0.018802 | 2.173691 | 0.043691 | 1.527528 |
| Gleem | 0.640721 |  | 0.223354 | 0.461751 | 1.467753 | 0.588348 | 1.807020 |
| Sidi bashr | 0.803580 | 0.223354 |  | 0.612234 | 1.135976 | 0.747329 | 1.874265 |
| Stanly | 0.018802 | 0.461751 | 0.612234 |  | 1.628282 | 0.013731 | 1.310741 |
| E.H. | 2.173619 | 1.467753 | 1.135976 | 1.628282 |  | 1.987538 | 1.453290 |
| El Mex | 0.043691 | 0.588348 | 0.747329 | 0.013731 | 1.987538 |  | 2.876512 |
| DK | 1.527528 | 1.807020 | 1.874265 | 1.310741 | 1.453290 | 2.876512 |  |

| Depend.:  β-carotene | Multiple Comparisons p values (2-tailed): β-carotene (Spreadsheet1) Independent (grouping) variable: group Kruskal-Wallis test: H (6, N=51) =9.249200 p=.1600 | | | | | | |
| --- | --- | --- | --- | --- | --- | --- | --- |
|  | Abo Qir  R:28.469 | Gleem  R:24.500 | Sidi bashr  R:22.750 | Stanly  R:28.625 | E.H  R:19.614 | El Mex:  R=22.245 | DK  R=25.611 |
| Abo Qir |  | 1.000000 | 1.000000 | 1.000000 | 0.624409 | 1.000000 | 1.000000 |
| Gleem | 1.000000 |  | 1.000000 | 1.000000 | 1.000000 | 1.000000 | 1.000000 |
| Sidi bashr | 1.000000 | 1.000000 |  | 1.000000 | 1.000000 | 1.000000 | 1.000000 |
| Stanly | 1.000000 | 1.000000 | 1.000000 |  | 1.000000 | 1.000000 | 1.000000 |
| E.H. | 0.624409 | 1.000000 | 1.000000 | 1.000000 |  | 1.000000 | 1.000000 |
| El Mex | 1.000000 | 1.000000 | 1.000000 | 1.000000 | 1.000000 |  | 1.000000 |
| DK | 1.000000 | 1.000000 | 1.000000 | 1.000000 | 1.000000 | 1.000000 |  |

Table S29. Kruskal-Wallis test for lycopene of tested seaweeds along Alexandria coast.

| Depend.:  Lycopene | Multiple Comparisons z´ values: Lycopene (Spreadsheet 1) Independent (grouping) variable: site Kruskal-Wallis test: H (6, N=51) =.8.881105 p=.1804 | | | | | | |
| --- | --- | --- | --- | --- | --- | --- | --- |
|  | Abo Qir  R:26.688 | Gleem  R:21.333 | Sidi bashr  R:24.417 | Stanly  R:24.00 | E.H  R:22.654 | El Mex:  R=21.765 | DK  R=22.654 |
| Abo Qir |  | 0.864385 | 0.319090 | 0.323391 | 1.220739 | 0.893246 | 1.956357 |
| Gleem | 0.864385 |  | 0.393528 | 0.298505 | 0.425436 | 1.537204 | 2.337653 |
| Sidi bashr | 0.319090 | 0.393528 |  | 0.043421 | 0.747608 | 0.999033 | 1.984112 |
| Stanly | 0.323391 | 0.298505 | 0.043421 |  | 0.625260 | 0.926834 | 1.903002 |
| E.H. | 1.220739 | 0.425436 | 0.747608 | 0.625260 |  | 1.876540 | 1.564320 |
| El Mex | 0.893246 | 1.537204 | 0.999033 | 0.926834 | 1.876540 |  | 2.982345 |
| DK | 1.956357 | 2.337653 | 1.984112 | 1.903002 | 1.564320 | 2.982345 |  |

| Depend.:  Lycopene | Multiple Comparisons p values (2-tailed): Lycopene (Spreadsheet1) Independent (grouping) variable: group Kruskal-Wallis test: H (6, N=51) =8.881105 p=.1804 | | | | | | |
| --- | --- | --- | --- | --- | --- | --- | --- |
|  | Abo Qir  R:26.688 | Gleem  R:21.333 | Sidi bashr  R:24.417 | Stanly  R:24.00 | E.H  R:22.654 | El Mex:  R=21.765 | DK  R=22.654 |
| Abo Qir |  | 1.000000 | 1.000000 | 1.000000 | 1.000000 | 1.000000 | 1.000000 |
| Gleem | 1.000000 |  | 1.000000 | 1.000000 | 1.000000 | 1.000000 | 0.407510 |
| Sidi bashr | 1.000000 | 1.000000 |  | 1.000000 | 1.000000 | 1.000000 | 0.992111 |
| Stanly | 1.000000 | 1.000000 | 1.000000 |  | 1.000000 | 1.000000 | 1.000000 |
| E.H. | 1.000000 | 1.000000 | 1.000000 | 1.000000 |  | 1.000000 | 1.000000 |
| El Mex | 1.000000 | 1.000000 | 1.000000 | 1.000000 | 1.000000 |  | 1.000000 |
| DK | 1.000000 | 0.407510 | 0.992111 | 1.000000 | 1.000000 | 1.000000 |  |

Table S30. Comparison of essential amino acids requirements (mg/kg/day) with FAO/WHO/

UNU recommendations (2007).

| Amino acids  (AAs) | FAO/WHO/UNU, 2007  requirement | Brown seaweeds | Red seaweeds | Green seaweeds |
| --- | --- | --- | --- | --- |
|  | (mg/kg/day) | *P. boryana* | *P.capillacea* | *U. fasciata* |
| Essential amino acids (EAAs) |  |  | | |
| Histidine (His.) | 10 | 16.78 | 7.50 | 5.39 |
| Threonine (Thr.) | 15 | 16.39 | 12.70 | 10.98 |
| Valine (Val.) | 26 | 23.67 | 13.92 | 13.99 |
| Methionine (Meth.) | 15 | 16.85 | 12.21 | 8.53 |
| Isoleucine (Ileu.) | 20 | 22.83 | 21.16 | 11.07 |
| Leucine (Leu.) | 39 | 31.39 | 25.86 | 10.37 |
| Phenylalanine (Phe.) | 25 | 20.47 | 42.90 | 27.14 |
| Lysine (Lys.) | 30 | 12.00 | 20.90 | 13.54 |
